# Chimpanzees' behavioral flexibility, social tolerance, and use of tool-composites in a progressively challenging foraging problem

**DOI:** 10.1016/j.isci.2021.102033

**Published:** 2021-01-05

**Authors:** Rachel A. Harrison, Edwin J.C. van Leeuwen, Andrew Whiten

**Affiliations:** 1 Centre for Social Learning and Cognitive Evolution, and Scottish Primate Research Group, School of Psychology & Neuroscience, University of St Andrews, St Andrews KY16 9JP, UK; 2 Department of Ecology and Evolution, University of Lausanne, Lausanne 1015, Switzerland; 3 Behavioral Ecology and Ecophysiology Group, Department of Biology, University of Antwerp, Universiteitsplein 1, 2610 Wilrijk, Belgium; 4 Centre for Research and Conservation, Royal Zoological Society of Antwerp, K. Astridplein 26, B 2018 Antwerp, Belgium

**Keywords:** Biological Sciences, Animals, Ethology

## Abstract

Behavioral flexibility is a critical ability allowing animals to respond to changes in their environment. Previous studies have found evidence of inflexibility when captive chimpanzees are faced with changing task parameters. We provided two groups of sanctuary-housed chimpanzees with a foraging task in which solutions were restricted over time. Initially, juice could be retrieved from within a tube by hand or by using tool materials, but effective solutions were then restricted by narrowing the tube, necessitating the abandonment of previous solutions and adoption of new ones. Chimpanzees responded flexibly, but one group increased their use of effective techniques to a greater extent than the other. Tool-composite techniques emerged in both groups, but primarily in the more flexible group. The more flexible group also showed higher rates of socio-positive behaviors at the task. In conjunction, these findings support the hypothesis that social tolerance may facilitate the emergence and spread of novel behaviors.

## Highlights

• Chimpanzees adapted their tool use to a change in foraging constraints
• Groups differed significantly in the level of behavioral flexibility observed
• The more flexible group showed higher rates of socio-positive behaviors at the task
• Tool-composite techniques were observed, primarily in the more flexible group

## Graphical abstract

**Figure:**
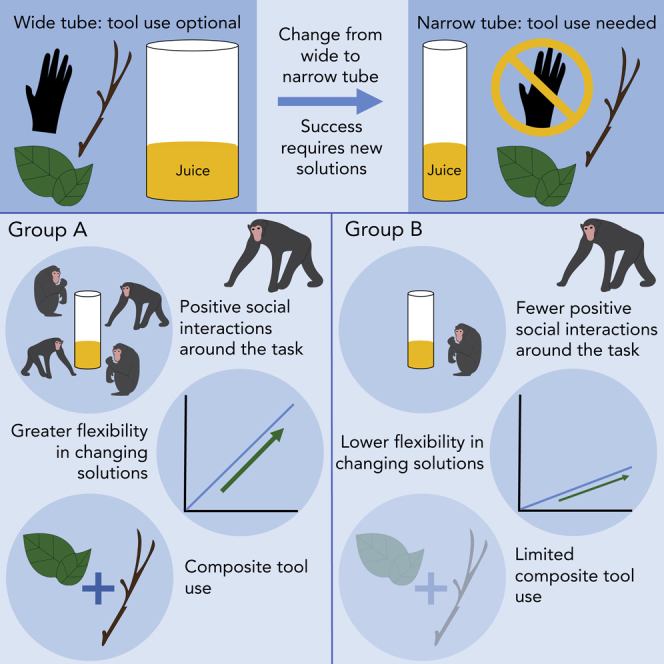

## Introduction

Behavioral flexibility is the ability to alter behavior following environmental feedback and to inhibit previously successful behaviors. It can allow organisms to adapt their behavior to suit changing or novel environments and supports problem solving (Griffin and Guez, 2014; Sol et al., 2002). It is, accordingly, a phenomenon of wide and general significance in understanding behavioral evolution. Behavioral flexibility has, however, with some justification been argued to be an ill-defined concept (Coppens et al., 2010; Audet and Lefebvre, 2017). Griffin and Guez (2014) suggested that behavioral flexibility is likely to encompass a range of abilities, including the ability to inhibit a previously rewarded behavior, to invent novel behavior, and to perform an existing behavior in a novel context. This analysis would suggest that behavioral flexibility may include capacities that are commonly described as innovation (defined as inventing novel behavior and performing existing behavior in a novel context by Kummer and Goodall, 1985) but in addition includes the ability to inhibit previously rewarded behavior.

Behavioral flexibility has been measured in a variety of ways, and different methods of assessing “behavioral flexibility” may in fact measure distinct cognitive capacities (reviewed by Audet and Lefebvre, 2017). One means of assessing a species' behavioral flexibility is through measuring the innovation rate (Sol et al., 2002; Nicolakakis et al., 2003; Sol and Lefebvre, 2000; Lefebvre et al., 1997, 2004) or the number of behavioral variants in a population (Wright et al., 2010) as proxy measures of flexibility. In experimental settings, reversal learning paradigms are frequently used, in which animals learn one association between stimuli, responses, and reward, and then in the test phase, reward contingencies are reversed (e.g., Manrique and Call, 2015). The speed at which an animal reverses its response preference in reversal learning experiments can accordingly be used as a measure of behavioral flexibility (Logan, 2016a), as can the frequency of errors during the test phase (Manrique and Call, 2015). However, some studies have found that reversal learning and innovation do not co-vary when tested under experimental conditions (Logan, 2016b; Griffin et al., 2013), suggesting that, although behavioral flexibility may facilitate innovation (Griffin and Guez, 2014), the relationship between the two may not be direct, and the findings of the latter suggest that the two abilities should be considered separately. Extractive foraging task paradigms, including multi-access boxes, are another approach that has been used to assess behavioral flexibility in non-humans (Auersperg et al., 2011; Lehner et al., 2011; Manrique et al., 2013; Richter et al., 2016; Davis et al., 2016; Harrison and Whiten, 2018). In these studies, animals can access food rewards by solving a physical problem, but once attained, this initial solution is rendered ineffective. Animals are thus challenged to find a novel solution, or modify the previously successful solution, in order to continue to retrieve rewards. Multi-access boxes require an animal to act on a new area of the apparatus, with a novel motor action (Auersperg et al., 2012), while some artificial foraging tasks may require the animal to modify a known behavior while acting upon the same part of the apparatus with similar motor actions (Lehner et al., 2011; Manrique et al., 2013; Harrison and Whiten, 2018). It is therefore possible that, within this category of extractive problem-solving tasks, different cognitive processes are being measured.

Such measures appear to call upon some of the abilities described by Griffin and Guez (2014) as components of behavioral flexibility, abandoning previously rewarded behaviors and applying novel behavior in response to novel conditions. Conceptually, there are clear similarities between this approach and classic reversal learning paradigms: once a rewarding behavior is attained, reward contingencies are altered such that previously rewarded behavior must be inhibited and novel behavior is displayed in order to attain a reward. However, Audet and Lefebvre (2017) argue that the direct relationship between cue and reward in a reversal task and the sudden and repeated changes in a cue's predictive value that characterize reversal and set-shifting tasks are not reflected in extractive foraging tasks in captivity or problem solving in the wild. Therefore, despite the surface-level conceptual similarity between these approaches to measuring behavioral flexibility, it remains possible that these tasks are measuring different processes.

Behavioral flexibility is thought to be key in supporting the evolution of cumulative culture, which underlies the unique complexity that human culture attains (Tomasello et al., 1993; Boyd and Richerson, 2005; Tennie et al., 2009; Dean et al., 2014). Cumulative culture is the process whereby individuals modify their socially learned behaviors, and these modifications to the behavior are retained in the population, in a process sometimes referred to as “ratcheting,” because modifications are retained with minimal loss or backward slippage (Tomasello, 1994; Tennie et al., 2009). Cumulative culture is distinct from mere cultural change, and from the accumulation of novel behaviors in a population's behavioral repertoire, in that it describes a process of repeated modification and transmission of traits, with these cultural traits increasing in their complexity, efficiency, or adaptiveness (Dean et al., 2014; Mesoudi and Thornton, 2018). This cumulative process allows the development, over time and transmission episodes, of behaviors and technologies more complex than any one individual could invent within their lifetime. In a cumulative process, modified and improved behaviors replace previous versions that are already present in a population's repertoire, and therefore, cumulative culture necessitates behavioral flexibility both in the process of innovative modification by some individuals and in the acquisition by others of these behaviors. Innovating entirely novel behaviors “from scratch” alone is not sufficient to support cumulative culture, which instead requires an ability to modify known behaviors (Charbonneau, 2015). Experimental research in humans has investigated the underlying processes that may facilitate cumulative culture, such as high-fidelity social learning (Caldwell and Millen, 2009; Derex et al., 2013), innovation (McGuigan et al., 2017; Miu et al., 2018, 2020), language (Morgan et al., 2015), and prosociality (Dean et al., 2012). Experimental, ethnographic, and archaeological studies highlight a potential role of demographic factors such as population size (Powell et al., 2009; Kline and Boyd, 2010; Derex et al., 2013) and connectivity (Hill et al., 2014; Derex and Boyd, 2016).

Evidence for cumulative culture in non-human animals, however, is extremely limited (Dean et al., 2014), although potential examples of cumulation have been put forward in chimpanzees (Boesch, 2003; Boesch et al., 2020), New Caledonian crows (Hunt and Gray, 2003), and Japanese macaques (Schofield et al., 2018). Behavioral flexibility, along with other components such as innovation (Bandini and Harrison, 2020) and high-fidelity social learning (Whiten, 2017; Boesch et al., 2020), should be considered and investigated as potentially limiting factors for cumulative culture in non-humans. When considering behavioral flexibility in the context of cumulative culture, the use of artificial extractive foraging tasks to assess an animal's ability to inhibit previously learned solutions and modify known behaviors seems a relevant and useful measure, as it bears a close resemblance to many types of problems dealt with by culturally transmitted behaviors in the wild.

In a study using an artificial extractive foraging task paradigm, Manrique et al. (2013) provided all four non-human great ape species with a puzzle box from which a food reward could be retrieved. The required solution changed over time, such that individuals had to repeatedly innovate in order to solve the task, and individuals were tested in isolation. All species, with the exception of orangutans (*Pongo abelii*) were able to solve all three stages of the task, setting aside obsolete techniques in order to do so. In other studies, individuals were given the opportunity to learn successful or more rewarding techniques socially (Yamamoto et al., 2013; Davis et al., 2016). For example, Davis et al. (2016) found that captive chimpanzees, trained to use a laborious method to solve an artificial foraging task, were able to switch to a more efficient method after observing a conspecific demonstrator. Using an artificial liquid-retrieval task in which solutions were restricted over time, Lehner et al. (2011) found that orangutans were capable of flexibly adjusting their behavior in response to task changes and, in addition, tool-composite techniques (the simultaneous use of two tools to achieve a single outcome, Shumaker et al., 2011) emerged, which the authors argue were cumulative combinations of previous techniques.

More generally, results have been mixed regarding chimpanzees' capacity for behavioral flexibility, with some studies finding that chimpanzees did not alter their behavior even when persistence with a known behavior resulted in no reward at all (for example, the “rattle specialists” in Hrubesch et al., 2009) or resulted in receiving a less valuable reward (Marshall-Pescini and Whiten, 2008). Others have found that chimpanzees are capable of altering their behavior to maximize rewards (e.g., van Leeuwen et al., 2013), although it has been suggested that this may only occur when their known solution becomes sufficiently inefficient relative to a novel solution (Davis et al., 2016), when the task in question is both simple and transparent enough to be causally understood (Jacobson and Hopper, 2019), or when their known solution is relatively simple and did not require significant investment to acquire (Davis et al., 2019).

The existing experimental evidence regarding behavioral flexibility in chimpanzees is thus quite divergent, likely due to differences in experimental paradigms, although we note observations indicating that individual chimpanzees can and do behave flexibly in certain ways in the wild (for example, an immigrant female chimpanzee was observed to adjust her choice of tools for nut cracking to match her new group, Luncz and Boesch, 2014). Further research is therefore required to shed light upon the situations in which chimpanzees may be able and motivated to abandon known techniques in favor of novel ones.

The current study investigated chimpanzees' capacity for behavioral flexibility when experimental conditions required behavioral alteration for the continued gaining of rewards. The use of such a changing, artificial foraging task to test for flexibility allows discussion of results in the context of the existing literature, much of which has used this approach (Hrubesch et al., 2009; Lehner et al., 2011; Manrique et al., 2013; Davis et al., 2016; Harrison and Whiten, 2018; Jacobson and Hopper, 2019). In the light of the work reviewed above, such studies may have significant implications for comparative research on cumulative culture. If chimpanzees are found to have the ability to behave flexibly under certain conditions, such conditions might also be those that can promote the emergence of cumulative technology.

### Study aims

In the present study, we aimed to expand upon the findings of our previous study with zoo-housed chimpanzees (Harrison and Whiten, 2018) by providing the same liquid-retrieval task, inspired by that used by Lehner et al. (2011) with orangutans, to two groups of chimpanzees (“Group 3”, N = 10, with N = 9 participating in the current study; “Group 4,” N = 12) housed in the Chimfunshi Wildlife Orphanage (CWO), a sanctuary in Zambia. In this “dipping” task, individuals could retrieve juice from within a tube using either their hands or provided tool materials. After 10 h, the width of the tube was narrowed (swapping from an initial “Wide Tube” phase to a “Narrow Tube” phase, presented for a further 20 h). This restricted the solutions available and necessitated tool use to retrieve the juice. Conducting the study at the CWO offered the opportunity to include infants, juveniles, and subadults in the sample. As age has been reported as a factor affecting both the acquisition of tool-based foraging behavior (Matsuzawa, 1994, 2007; Biro et al., 2006, 2010) and cognitive flexibility in chimpanzees (Manrique and Call, 2015), we anticipated that this would increase the probability of greater levels of flexibility and innovation. In addition, the CWO offered the opportunity to test multiple groups housed in identical conditions, allowing us to conduct between-group comparisons and explore group-level factors, such as social tolerance, a factor previously shown to differ between the two groups we tested at the CWO (Cronin et al., 2014a), that might underlie performance on this type of artificial foraging task. Chimpanzees at the CWO had not participated in any other tool-use-based experimental task prior to the data collection period of this study. Group 4 had previously participated in a token-exchange study (van Leeuwen et al., 2013).

### Behavioral flexibility

Chimpanzees were presented with the task in the social environment of their group, providing a naturalistic context in which they could obtain information about the task and potential solutions both by means of individual and social learning. This means that individuals were not limited to what they themselves could invent in terms of task solutions and could also observe solutions used by others. We believe this provides an ecologically valid measure of behavioral flexibility, as this has been defined in the past as the continued acquisition of new solutions through either innovation or social learning (Lehner et al., 2011). If chimpanzees are capable of responding flexibly to changes in task conditions (as shown by Manrique et al., 2013), we predicted the individuals in this study would increase their use of techniques that remained effective, and decrease their use of techniques that had been rendered ineffective, in the face of task restrictions. Alternatively, the continued use of ineffective techniques and no increase in the use of effective techniques by the chimpanzees in this study would lend further evidence to an argument that chimpanzees are generally relatively behaviorally inflexible. The task is cumulatively challenging in nature, in that some restrictions required not only a change in behavior from the chimpanzees but also the modification of known behaviors in order to continue to gain rewards.

### Scaffolding toward an effective solution

Based upon their performance in the first two phases of the study (i.e., the Wide Tube phase followed by a Narrow Tube phase), we provided one group of chimpanzees with scaffolding (providing individuals with the physical artifacts of tool use to interact with, as chimpanzees are likely to experience in the wild) potentially facilitating a behavior (tool-composite use) previously observed in their group but performed infrequently by a very limited number of individuals. The scaffolding was provided only to one group (Group 3), as tool-composite use was already well established in the other group (Group 4) by the end of the Narrow Tube phase. Introducing scaffolding increased the (so far) limited number of experimental investigations into this subject. Although it has been suggested that scaffolding may be a significant route through which chimpanzees acquire tool use in the wild (Tennie et al., 2009; Fragaszy et al., 2013), studies to date have not found that it leads to the acquisition of novel behaviors (Gruber et al., 2011; Cardoso and Ottoni, 2016; Harrison and Whiten, 2018). The presence of social information in the current study (owing to some individuals infrequently performing the target behavior) was predicted to increase the probability of scaffolding proving effective, as it could be bolstered by social observation of an individual performing the behavior. Therefore, the scaffolding in the current study was expected to function in a manner more similar to the artifacts chimpanzees encounter in the wild, where such artifacts are only part of the social information an individual has access to when acquiring a new behavior (Fragaszy et al., 2013).

### Social tolerance and behavior at the task

Providing the foraging task in a group context also allowed us to analyze the social behaviors and interactions exhibited by individuals at the task. Social behaviors at the task were measured in order to obtain an insight into the social tolerance of both groups, and the behaviors measured (aggression, displacement, co-action, concurrent action, peering, scrounging, and tool transfer; see Transparent methods for definitions) also had the potential to impact directly upon task performance by influencing the likelihood of social learning and innovation during the task. Social tolerance has been defined as the propensity to “be in proximity to conspecifics around valuable resources with little or no aggression” (Cronin and Sánchez, 2012, p.4). High social tolerance can facilitate the social learning of foraging behaviors, as it allows for proximity during foraging (permitting observation of behaviors), reduces the likelihood of antagonistic interactions (again, permitting uninterrupted observation of behaviors), and allows subordinate animals to retain any food acquired (Coussi-Korbel and Fragaszy, 1995; van Schaik, 2003). van Schaik (2003) argues that, as well as facilitating social learning, social tolerance may also promote innovation, with increased opportunities for uninterrupted object manipulation being provided. Previous research on the same groups of captive chimpanzees studied here (Cronin et al., 2014a; van Leeuwen et al., 2018) has found that the groups have differing patterns of sociality, with one group (Group 4) appearing more socially tolerant than the other (Group 3). These studies used social network analyses and experimental measures of co-feeding tolerance. Our study explores whether apparent differences in the groups' social tolerance influenced their social behaviors at and around an artificial foraging task, thus allowing us to assess whether, in proximity to this valuable resource, either group showed more aggressive or more tolerant behaviors. Social tolerance could conceivably impact the performance on the current task in a variety of ways. One possibility is that socially tolerant individuals possess cognitive characteristics that promote behavioral flexibility. Another possibility is that social tolerance promotes social learning as tolerance allows closer proximity to other foraging individuals, providing opportunities for observational learning (van Schaik, 2003), which in this task could aid individuals in acquiring new task solutions for use in the Narrow Tube phases. Lastly, social tolerance may lead to individuals gaining uninterrupted access to the task more easily by making displacement by others less likely, perhaps facilitating innovation (van Schaik, 2003) by allowing individuals to discover effective solutions in the Narrow Tube phases.

## Results

### Behavioral flexibility in the use of Always Effective techniques

**Figure 1:**
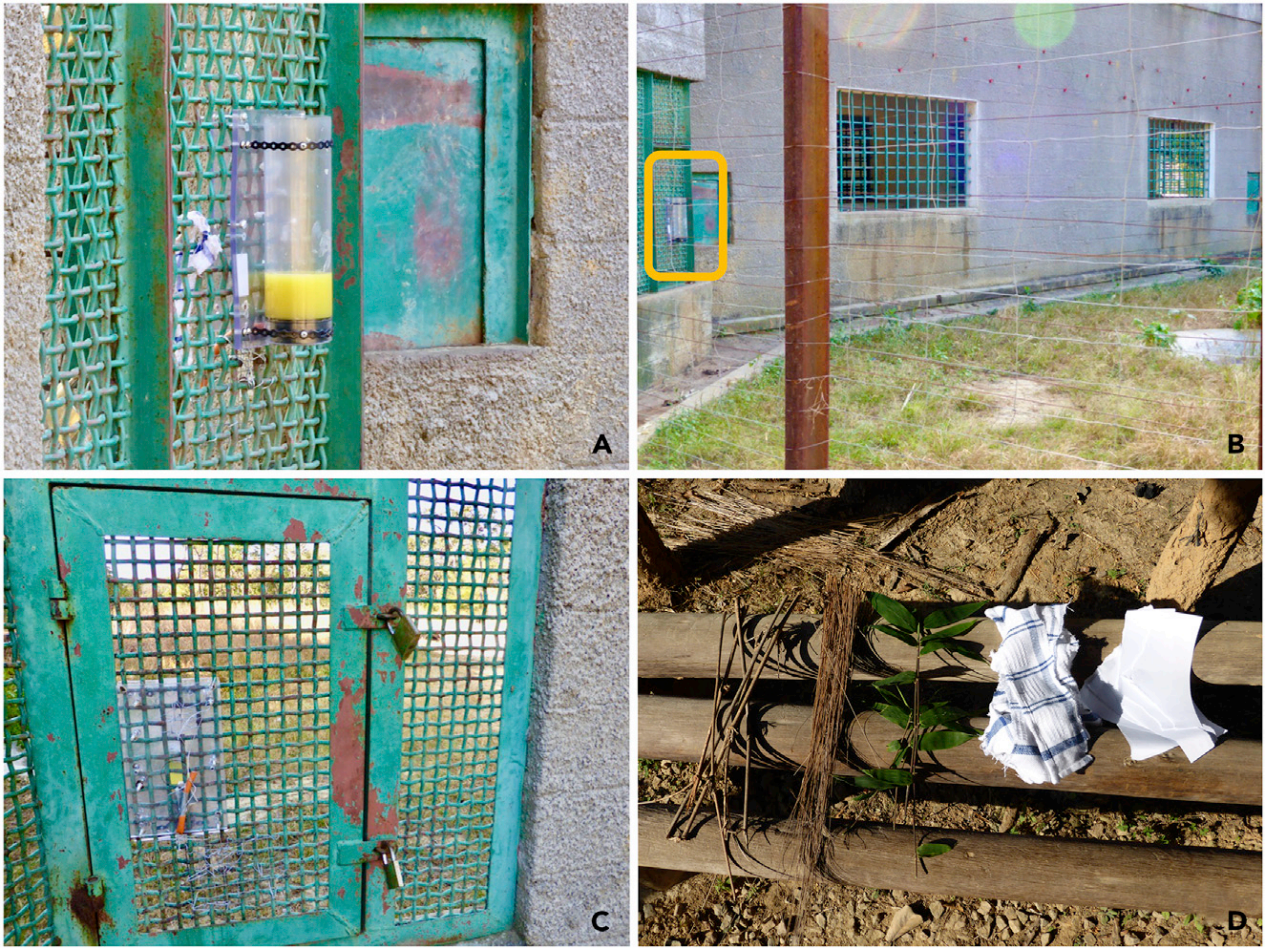
Experimental setup (A) The “wide” tube attached to the mesh door in Group 4's enclosure and filled to around 7 cm with diluted juice. (B) The wider context in which the tube was located, with the tube (circled in yellow) attached to a mesh door forming part of the chimpanzees' indoor facility. (C) The experimenter's view of the task when fitted, facing out into the chimpanzees' enclosure. (D) The array of tool materials provided to the chimpanzees (from left, plain sticks, straw, leafy sticks, strips of cloth, and strips of paper).

Chimpanzees in two sanctuary-housed groups (named Group 3 and Group 4, see Transparent methods for group composition) were presented with an artificial foraging task (see Figure 1) in which juice could be retrieved from a tube either by hand or using provisioned tool materials (the Wide tube phase, Figure 1A).

After 10 h of exposure, the tube was replaced with a narrower version, restricting the solutions available (narrow tube phase, presented for 20 h). Attempts made by individuals were coded as “Always Effective” or “Initially Effective” based upon their efficacy across the Wide and Narrow Tube phases, allowing analysis of whether chimpanzees altered their behavior to respond to the change in task parameters. Techniques involving the insertion of a hand into the tube were classified as Initially Effective, as while they worked in the first, Wide Tube, phase, insertion of the hand into the tube was made impossible by the width of the tube in the Narrow Tube phase. Techniques that did not involve insertion of a hand into the tube were classified as Always Effective, as they had the potential to be used successfully in both the Wide and Narrow Tube phases (see Tables 1 and 2). This designation does not assume such techniques necessarily to be efficient or to have particularly high success rates, as techniques classified as Always Effective could be challenging to perform in terms of manual skill (e.g. *stick push and retrieve* techniques) and thus have a relatively low success rate (although success would still be higher than the Initially Effective techniques that were generally rendered impossible by task constraints in the Narrow Tube phase) or such techniques could be time consuming to perform and therefore be relatively inefficient. Similarly, Initially Effective techniques could on rare occasion be used successfully in the Narrow Tube phases (for example, if a large amount of material is built up in the tube during a session, it could be possible to successfully use techniques such as *sugarcane retrieve*). However, use of these Initially Effective techniques, which relied upon inserting a hand into the tube, indicated continuing use of an approach that was not well suited to the task constraints.

**Table 1:** Techniques used successfully by Group 3 to solve the task

Technique	Description	Time to first occurrence from start of testing (hh:mm:ss)	Efficacy	First successfully performed by: ID (sex, age)
Wide tube phase				
Cloth dip	Dip cloth into juice with hand	00:01:28	Initially Effective	Barbie (F, 20)
Cloth drop	Drop cloth from hand into tube and retrieve	00:01:38	Initially Effective	Barbie (F, 20)
Hand dip	Dip hand directly into juice	00:02:10	Initially Effective	ET (F, 20)
Cloth mouth drop	Drop cloth from mouth into tube then retrieve by hand	00:03:26	Initially Effective	Clement (M, 22)
Cloth retrieve	Cloth that has accrued in tube removed with hand	00:29:32	Initially Effective	Bruce (M, 5)
Fruit stone mouth drop	Drop fruit stone from mouth into juice then retrieve by hand	03:21:25	Initially Effective	Bruce (M, 5)
Sugarcane drop	Drop sugarcane from hand into tube and retrieve	05:54:53	Initially Effective	Lods (F, 5)
Sugarcane dip	Dip sugarcane into juice with hand	05:55:06	Initially Effective	Lods (F, 5)
Sugarcane retrieve	Sugarcane that has accrued in tube removed with hand	05:56:02	Initially Effective	Lods (F, 5)
Sugarcane mouth drop	Drop sugarcane from mouth into tube then retrieve by hand	08:46:26	Initially Effective	Lods (F, 5)
Narrow tube phase				
Stick dip	Dip stick into juice with hand	13:19:21	Always Effective	ET (F, 20)
a Stick retrieval paper	Stick used to retrieve paper that has accrued in tube	13:20:53	Always Effective	ET (F, 20)
Stick retrieve	Stick already in tube removed with hand	13:21:13	Always Effective	ET (F, 20)
a Stick retrieval cloth	Stick used to retrieve cloth that has accrued in tube	13:27:39	Always Effective	ET (F, 20)
Narrow scaffolded phase				
Stick drop	Drop stick into tube then retrieve by hand	32:09:22	Always Effective	Roxy (F, 20)

a Tool-composite technique.

**Table 2:** Techniques used successfully by Group 4 to solve the task

Technique	Description	Time to first occurrence from start of testing (hh:mm:ss)	Efficacy	First successfully performed by
Wide tube phase				
Paper dip	Dip paper into juice with hand	00:00:31	Initially Effective	Nicky (M, 24)
Paper drop	Drop paper from hand into tube and retrieve	00:03:12	Initially Effective	Nicky (M, 24)
Paper retrieve	Paper that has accrued in tube removed with hand	00:04:16	Initially Effective	Nicky (M, 24)
Cloth dip	Dip cloth into juice with hand	00:11:04	Initially Effective	Nicky (M, 24)
Cloth retrieve	Cloth that has accrued in tube removed with hand	00:12:59	Initially Effective	Nicky (M, 24)
Cloth drop	Drop cloth from hand into tube and retrieve	00:13:09	Initially Effective	Nicky (M, 24)
Hand dip	Hand dipped directly into juice	00:14:40	Initially Effective	Nicky (M, 24)
Cloth mouth drop	Cloth dropped from mouth into tube then retrieved with hand	00:44:08	Initially Effective	Nicky (M, 24)
Paper mouth drop	Paper dropped from mouth into tube then retrieved with hand	01:34:03	Initially Effective	Nicky (M, 24)
Stick drop	Drop stick from hand into tube and retrieve	05:23:54	Always Effective	Bobby (M, 22)
Stick dip	Dip stick into juice with hand	05:24:05	Always Effective	Bobby (M, 22)
Fruit shell drop	Drop fruit shell from hand into tube and retrieve	08:10:03	Initially Effective	Nicky (M, 24)
Narrow tube phase				
Stick retrieve	Stick already in tube removed with hand	10:16:56	Always Effective	Jack (M, 7)
a Stick retrieval paper	Stick used to retrieve paper that has accrued in tube	10:26:23	Always Effective	Kathy (F, 16)
a Stick retrieval stick	Stick used to retrieve stick already in tube	10:28:57	Always Effective	Kathy (F, 16)
a Stick push cloth and retrieve	Stick used to push cloth down into juice and then retrieve by pushing cloth against wall of tube and pulling upwards	14:17:49	Always Effective	Jack (M, 7)
a Stick retrieval cloth	Stick used to retrieve cloth that has accrued in tube	15:03:10	Always Effective	Kathy (F, 16)
Sugarcane dip	Sugarcane is dipped directly into juice with hand	19:09:29	Initially Effective	Jack (M, 7)
Sugarcane retrieve	Sugarcane that has accrued in tube removed with hand	19:25:56	Initially Effective	Jack (M, 7)
a Stick retrieval sugarcane	Stick used to retrieve sugarcane that has accrued in tube	19:27:52	Always Effective	Jack (M, 7)
a Stick push sugarcane and retrieve	Stick used to push sugarcane down into juice and then retrieve by pushing sugarcane against wall of tube and pulling upwards	19:35:51	Always Effective	Kathy (F, 16)
a Cloth drop stick retrieve	Cloth dropped from hand into tube then retrieved with stick	22:48:25	Always Effective	Kit (M, 10)
Narrow restricted tube phase				
a Stick push straw and retrieve	Stick used to push straw down into juice and then retrieve by pushing straw against wall of tube and pulling upwards	30:13:18	Always Effective	Jack (M, 7)
a Stick retrieval plastic wrapper	Stick used to retrieve plastic wrapper already in tube	30:56:56	Always Effective	Jack (M, 7)

a Tool-composite technique.

In comparison with an analysis based upon the rate of use of specific techniques (e.g., *stick dip* or *hand dip*), this approach of classifying attempts into Always Effective or Initially Effective avoids potential confounds such as individual differences in skill or preference and also allows for potential changes in the availability of tool materials over the course of a session (as Always Effective techniques were always achievable for individuals even if specific tool materials were depleted). The techniques used, the point in the study at which they emerged, their categorization as either Always Effective or Initially Effective during the Narrow Tube phase, and the identity, age, and sex of the first individual to successfully use the technique are shown in Tables 1 and 2. Note that the techniques involving the use of sugarcane, provided as part of the chimpanzees' normal diet, describe instances in which the internal fibrous part of the sugarcane was chewed and then used as an absorbent material.

**Figure 2:**
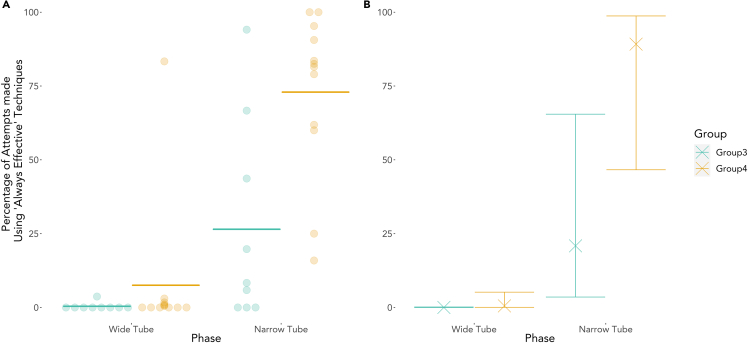
Change in use of effective techniques across Wide and Narrow Tube conditions (A) Observed percentages of “Always Effective” (for narrow tube) technique use in the “Wide” and “Narrow” tube phases. Points show the observed percentage of attempts made using Always Effective techniques by individual chimpanzees. Observed means are shown by solid horizontal bars. (B) Predicted percentages, based upon the full model for Groups 3 and 4. Predicted values from the model for each group in each phase are shown by bold crosses. Error bars show 95% confidence interval of these predicted values. See also Figure S1.

Only one individual (Bobby, Group 4) used Always Effective techniques for the majority of his attempts in the Wide Tube phase (5 of 6 attempts, see Figure S1), with all the other individuals in both groups using Always Effective techniques for fewer than 4% of their attempts in the Wide Tube phase (see Figure 2). Therefore, the imposition of the narrow tube and the resulting necessity of the use of Always Effective techniques for success represented a genuine restriction upon the behavior of almost all individuals relative to their earlier repertoire. Fourteen individuals (eight in Group 3, six in Group 4) had interacted with the task during the Wide Tube phase but had never used a technique that would remain effective in the Narrow Tube phase before the introduction of the narrow tube.

We conducted a binomial GLMM with logit link function to study the effects of Phase, Group, Age, and Sex on the probability to use Always Effective techniques (yes/no). A likelihood ratio test comparing the full and null models (see Transparent methods for further details) indicated that the full model was a significantly better fit (χ^2^ = 48.72, df = 4, p <.0001; dAIC = 40.8). The full statistical model (see Table 3) indicated a significant effect of Phase (LRT: χ^2^ = 38.26, p < 0.001), such that the odds for individuals to use Always Effective techniques in the Narrow Tube phase were 1,525.38 (95% confidence interval [CI] [372.41, 27,722.51]) times larger than in the Wide Tube phase. Moreover, the odds for individuals in Group 4 to use Always Effective techniques were 31.19 (95% CI [2.16, 83.93]) times larger than for individuals in Group 3 (LRT: χ^2^ = 8.22, p = 0.004). No effect of either Sex or Age was found (Sex - LRT: χ^2^ = 0.28, p = 0.60; Age - LRT: χ^2^ = 0.00, p = 0.99). See Table 3 for the full model parameters and Figure 2B for predicted values plus 95% CIs based upon the full model. Individuals in Group 4 used Always Effective techniques for a mean of just 7.5% (SD = 23.9) of their attempts in the Wide Tube phase, but this increased to a mean of 72.9% (SD = 27.7) of their attempts made in the Narrow Tube phase (note that these group means are based upon the percentage of Always Effective attempts each individual made in a given phase). Group 3 used Always Effective techniques for a mean of only 0.4% (SD = 1.2) of their attempts in the Wide Tube phase and for a mean of 26.5% (SD = 34.3) of their attempts in the Narrow Tube phase (see Figure 2A). No individual in Group 3 had successfully used an Always Effective technique during the Wide Tube phase.

**Table 3:** Results of full model GLMM on the effects of Phase, Group, Age, and Sex upon “Always Effective” technique use

	Estimate	Wald 95% CI	Std. Error	z Value	p Value
(Intercept)	−8.66	−11.74, −6.81	1.20		
Phase (narrow)	7.33	5.92, 10.23	0.68	10.77	<0.001∗∗∗
Group (4)	3.44	0.77, 4.43	1.21	2.84	0.005∗∗
Age	0.01	−0.97, 0.99	0.50	0.02	0.99
Sex (male)	−0.63	−2.98, 1.72	1.20	−0.53	0.60

∗∗∗p < 0.001, ∗∗p < 0.01.

### A group difference in the use of Always Effective techniques

The main effect of Group found in the generalized linear mixed models (GLMM) analysis suggests that Group 4 was more likely to use Always Effective techniques throughout both Phases of the study, while visual examination of the data (Figure 2) suggests the possibility of an interaction between Group and Phase, in that Group 4 appeared to have used Always Effective techniques to a greater extent in Phase 2 compared with Phase 1 than did Group 3. The limited sample size, and variation in the extent to which individuals chose to participate in the study, precluded further investigation of this potential group difference using mixed modeling techniques. Instead, a post hoc non-parametric analysis was used to explore this effect. A Mann-Whitney U test comparing the increase in the proportion of Always Effective techniques used by each individual in Phase 2 relative to Phase 1 between the two groups indicated that individuals in Group 4 increased their use of Always Effective techniques to a greater extent than did individuals in Group 3 (Median increase in Group 3 = 8.3%, Median increase in Group 4 = 77.86%, Mann-Whitney *U* = 20, *n*_1_ = 9, *n*_2_ = 12, p = 0.017 two tailed).

### Tool-composite techniques: further restrictions versus scaffolding

Tool-composite techniques were observed in both groups, although to a greater extent (both in terms of frequency of use and the number of individuals performing the techniques) in Group 4 (see Tables 1 and 2). Following Shumaker et al. (2011), we defined tool-composite techniques as those in which two tool materials were used in combination to achieve a single goal. We note that this definition is at odds with the way composite tools are defined in the human-focused literature (as conjunctions of firmly connected units, such as hafted stone axes, Oswalt, 1976; Ambrose, 2010). We considered cases in which an individual used a stick to retrieve material that had previously accrued in the tube (e.g., *stick retrieval cloth*, *stick retrieval sugarcane*) to be examples of tool-composites, regardless of whether the material in the tube had previously been placed there by the individual performing the tool-composite behavior or another individual, as we considered materials within the tube to be tool materials. Note that some tool-composite behaviors observed during the study explicitly require the same individual to insert the absorbent material into the tube and retrieve it (e.g., *stick push cloth and retrieve, cloth drop stick retrieve*). After 20 h of exposure to the Narrow Tube phase, Group 4 had a subset of tool materials, primarily used in their existing tool-composite techniques, removed from their enclosure (“Narrow Restricted” phase; 10 h of exposure). Group 3 received the narrow tube with a stick and cloth already inserted (“Narrow Scaffolded” phase; 10 h of exposure) to potentially facilitate the adoption of this tool-composite technique.

In the Narrow Restricted phase, individuals in Group 4 showed some capacity to modify tool-composite techniques (see Table S2) by incorporating novel absorbent materials when preferred materials were no longer provided. However, it must be noted that such attempts were rare in the Narrow Restricted phase (0.89% of all attempts) while “*stick dip*” made up the majority (96.99%) of attempts made at the task in this phase. Thus, although chimpanzees appeared capable of modifying their known tool-composite techniques, they did not use these new solutions frequently, and these modified solutions most often proved to be unsuccessful (see Table S2). As known techniques (most notably, *stick dip*) remained possible during the Narrow Restricted phase, there was not the same necessity to behave flexibly as there was in the transition from Wide Tube to Narrow Tube phases.

Chimpanzees in Group 3 were exposed to the task with a stick and attached piece of cloth already inserted (Narrow Scaffolded phase), to explore the potential use of tool-composite techniques when scaffolded in this way, given the minimal prior innovations in this group. Some limited use of tool-composite techniques had been observed in Group 3 during the Narrow Tube phase, with two individuals both having successfully used sticks to retrieve cloth or paper from the tube, although only on a limited number of occasions. The scaffolded solution was presented at the beginning of Narrow Scaffolded sessions a total of six times. Three individuals interacted with the example solution, and these interactions are described in detail in Table S3. Only one individual attempted a tool-composite technique following exposure to the scaffolded solution, making four unsuccessful attempts to retrieve cloth that had accrued in the tube earlier in the day (28 min and 23 s following exposure to the example solution), although she had in previous sessions successfully retrieved cloth from the tube using a stick. Thus, it would appear that the provision of a scaffolded solution in this manner did not elicit the use of tool-composite techniques by individuals that had not already performed them.

### Group differences in social tolerance at the task

Given the apparent group differences in behavioral flexibility, and prior evidence of group differences in social tolerance (Cronin et al., 2014a; van Leeuwen et al., 2018), we conducted an exploratory analysis of social tolerance at the task. All video records from the task were coded with a specific focus upon social interactions relevant to social tolerance. Behaviors assumed to be “positive” and “negative” indicators of social tolerance were coded. The behaviors coded were aggression, displacement, co-action, concurrent action, peering, scrounging, and tool transfer. Definitions, and further discussion of the behaviors chosen as indicators of social tolerance, are provided in the Transparent methods and Supplemental information. The hierarchies of both groups were relatively stable at the time of testing, with no serious challenges to the position of the dominant male in each group. As Group 4 spent a greater amount of time at the task than Group 3, in order to compare the frequency of these social interactions between the groups, it is necessary to control for the amount of time during which the groups could be observed at the task. In Table 4, we present the frequency of each event type in terms of events per hour of observation for each group (the time for which at least one individual in a group was present at the task), along with the raw frequency of events observed in each group. For more detailed description of the social behaviors observed at the task (including discussion of which sex, age, and kin classes were more often involved in each type of social behavior coded), see Supplemental information.

**Table 4:** Social interactions in each group per observation hour and in total

“Positive” or “negative” indicator	Interaction type	Group 3		Group 4	
		Events per hour	Total number of events	Events per hour	Total number of events
Negative	Aggression	0.33	4	0.24	5
	Displacement	1.71	21	0.95	20
Positive	Co-action	0.16	2	0.62	13
	Concurrent action	0.41	5	1.05	22
	Peering	1.39	17	1.33	28
	Scrounging	0.08	1	1.14	24
	Tool transfer	0.33	4	1.10	23

In considering these behaviors as indicators of social tolerance, they can be grouped into likely positive indicators and likely negative indicators (with co-action, concurrent action, peering, scrounging, and tool transfer as positive indicators and aggression and displacement as negative indicators). Grouping the behaviors in this manner allows comparison of the difference in frequency of positive and negative indicators between the two groups. A chi-square test indicated that the two groups differed in terms of the proportion of positive and negative social interactions observed (χ^2^ (1) = 13.9, p < 0.001), with a greater proportion of positive indicators observed in Group 4.

## Discussion

Chimpanzees in both groups responded with a degree of flexibility to the changing artificial foraging task. Group differences were apparent in the responses, with individuals in Group 4 increasing their use of Always Effective techniques to a greater extent than Group 3. Tool-composite techniques were observed in both groups, but primarily in Group 4. Restricting the available tool materials in the Narrow Restricted phase presented to Group 4 resulted in some modification of these tool-composite techniques, but performance of these modified tool-composite techniques was limited. Providing Group 3 with scaffolding toward tool-composite techniques in the Narrow Scaffolded phase proved ineffective. Across the social behaviors recorded at and around the task, Group 4 had a higher rate of positive interactions than Group 3. Individuals in Group 4 were less likely to displace one another at the task and had a higher rate of tolerant interactions including those likely to aid in social learning, such as co-action and tool transfer.

### Group differences in behavioral flexibility

Both groups of chimpanzees responded with some level of flexibility to the change in task parameters, and the change in parameters represented a genuine restriction to both groups, as individuals in both groups (with only one exception, Bobby, in Group 4) had little use of or experience of techniques in the Wide Tube phase that would be effective in the Narrow Tube phase. Overall, the use of Always Effective techniques (that is, techniques that could be used effectively in both the Wide and Narrow Tube phases) increased significantly in the Narrow Tube phase, when they became necessary for success. However, this adaptive adjustment of techniques was not equally present in the two study groups. The majority of chimpanzees in Group 3 continued to primarily use Initially Effective techniques in the Narrow Tube phase, indicating a failure to relinquish these solutions and therefore somewhat more limited behavioral flexibility than chimpanzees in Group 4. Most chimpanzees in Group 4 primarily used Always Effective techniques in the Narrow Tube phase, indicating more pronounced behavioral flexibility in that they not only used novel solutions in response to the change in task but were also able to relinquish Initially Effective techniques (which they used predominantly in the Wide Tube phase) to a greater extent than individuals in Group 3. As this comparison was made across the Wide and Narrow tube phases only, in which task presentation was the same for both groups, an examination of the possible factors promoting these group differences is warranted.

Although the fact that only two groups were tested in the current study limits the extent to which firm conclusions can be drawn regarding what factors may have driven the group-level difference in flexibility observed, similarities between the two groups make it possible to rule out certain explanations with some confidence. The two groups live in near-identical enclosures and do not differ systematically in either rearing history or subspecies. Neither group had participated in any tool-use study prior to the current study (personal communication, Chimfunshi Research Advisory Board). We have no evidence suggesting a difference in prior naturally occurring tool-use between the two groups (see Table S4 for observational data collected by R.A.H. before beginning the current study), although it remains possible that the chimpanzees use tools more frequently in the forested areas of their enclosures, which cannot be observed. The two groups also do not differ greatly in terms of age distribution, with both groups including infants, juveniles, and adults (and our analysis indicates that age differences do not contribute to explaining the levels of behavioral flexibility observed).

One previously documented difference between the groups is in their level of social tolerance, with Group 4 more socially tolerant than Group 3 (Cronin et al., 2014a; van Leeuwen et al., 2018). The current study found differences between the two groups in the frequency of positive and negative social interactions at the artificial task. Group 4, which performed most flexibly at the task, also evidenced more social tolerance in their interactions at the task, with lower rates of displacement and higher rates of co-action, tool transfer, and tolerated scrounging. This meets the expectations of a socially tolerant group following van Schaik's (2003) hypotheses that social tolerance facilitates social learning and facilitates innovation by reducing the risk of displacement. Group 4 showed higher rates of tolerant behaviors at the task, and the occurrence of behaviors such as tool transfer and concurrent action (or task sharing) between unrelated individuals, including within dyads involving a non-kin adult male (see Supplemental information for more details), also indicates a level of social tolerance within this group.

Displacements occurred less frequently in Group 4 than in Group 3, a finding that is in line with van Schaik's (2003) hypothesis that social tolerance can impact positively upon innovation by reducing the likelihood of displacement. This may have allowed individuals in Group 4 to attempt the task without interruption, facilitating innovation (and indeed, as a group, more solutions were observed in Group 4 than in Group 3). It is also possible that, by chance, Group 4 contained more innovative individuals, as innovation propensity can vary between individuals (Amici et al., 2019). In order to disentangle whether individuals in Group 4 were more likely to innovate for intrinsic reasons (i.e., differences in personality, such as increased neophilia or persistence, Amici et al., 2019), or whether individuals in this group were more likely to innovate owing to decreased risk of displacement, a study would be required comparing rates of innovation between the groups in both social and individual testing conditions. The two possibilities are, of course, not mutually exclusive; Group 4 may have contained more innovative individuals who additionally benefited from a decreased risk of displacement. The potential role of social learning should also be considered. A decreased risk of displacement may have facilitated the spread of techniques via social learning, as individuals could observe one another without displacing each other. The higher rate of concurrent action in Group 4 compared with Group 3 also indicates that individuals in Group 4 were able to attempt the task while in close proximity to one another, and even to attempt the task simultaneously, without one individual monopolizing it. The capacity for some individuals at the CWO to attempt a task concurrently was previously documented by Cronin et al. (2014b). Of interest, during their study, Kathy was a member of two of the dyads that shared space on the “tower apparatus” (see Cronin et al., 2014b) most frequently, and in the current study, Kathy was the most frequent participant in concurrent action. This raises the possibility that individual differences in social tolerance may have important implications in experimental studies, across a range of paradigms. Although group-level differences in social tolerance shown in this study via descriptive data broadly follow the group-level difference in behavioral flexibility, more research is needed to explore the potential relationships between the two, ideally incorporating multiple measures for tolerance and flexibility at both an individual and group level across multiple groups.

Many studies of behavioral flexibility in chimpanzees examine only one group of chimpanzees (Bonnie et al., 2012; Manrique et al., 2013; Hopper et al., 2015a). The findings of this study indicate that this approach may not lead to generalizable results, as the two groups in our study, despite sharing identical environments and similar rearing histories, performed quite differently (see Kaufhold and Van Leeuwen, 2019). Our examination of social interactions at the task highlights the fact that group-level, intraspecific differences in social tolerance may impact social behavior in a way that impacts performance on artificial tasks, particularly if these tasks are presented in group contexts (e.g., Lehner et al., 2011). Damerius et al.’s (2017) finding that captive orangutans living in larger social groups were more curious and performed better on a cognitive test battery implies that differences in captive primates' social environments may also affect their performance when tested individually. Further research is required in order to tease apart the mechanisms by which differences in social tolerance might impact either performance on artificial foraging tasks (for example, by examining whether differences in rates of behaviors such as co-action and tool transfer lead to enhanced social learning of task solutions) or cognition directly (by examining whether individuals in more tolerant groups continue to show enhanced task performance when tested individually, as Damerius et al., 2017, finding might lead one to suspect). This finding has broader implications that groups differ in their propensity for behavioral change, perhaps owing to factors such as social tolerance that can promote social learning and innovation, and thus may also differ in their capacity for cumulative change.

The limited flexibility shown by Group 3 appears to concur with the results of several previous studies of chimpanzee behavioral flexibility in response to artificial foraging tasks (Marshall-Pescini and Whiten, 2008; Hrubesch et al., 2009; Harrison and Whiten, 2018), in which chimpanzees continued to use ineffective or inefficient techniques in response to task changes. In Hrubesch et al. (2009), some chimpanzees continued to attempt a task solution (rattling a board holding food, rather than manipulating the food items with a stick) that had been rendered completely ineffective by task manipulations, whereas in Marshall-Pescini and Whiten (2008), chimpanzees failed to acquire a more effective solution they witnessed. The results of the current study are perhaps closer in character to those of Hrubesch et al. (2009), as techniques the chimpanzees had mastered in the Wide Tube phase were rendered ineffective in the Narrow Tube phase. The results from this group therefore appear to add to a literature in which chimpanzees show only limited evidence of the capacity to respond flexibly to task changes in artificial foraging tasks. However, in light of our findings regarding social tolerance at the task, it is unclear to what extent chimpanzees in Group 3 had less capacity for behavioral flexibility, and to what extent their ability to demonstrate that flexibility was impeded by a lack of social tolerance (resulting in an increased risk of displacement and reduced opportunities for social learning) in comparison with Group 4.

In the Wide Tube phase, chimpanzees in Group 3 did not successfully use a solution to the task that would remain effective in the Narrow Tube phase. The resulting necessity for innovation in the Narrow Tube phase may have contributed to their apparently limited flexibility, and indeed, they did not successfully use an Always Effective technique until 3 h of the Narrow Tube phase had passed (whereas in Group 4, one individual had used an Always Effective technique in hour 5 of the Wide Tube phase). The necessity to invent novel solutions to a problem may be an additional challenge in artificial foraging tasks aiming to assess behavioral flexibility, and one that may not be shared by measures such as reversal learning, or indeed, studies in which novel techniques can be socially learned (e.g., Marshall-Pescini and Whiten, 2008; Davis et al., 2016). However, the chimpanzees in Group 3 invented 15 task solutions, all of which were successfully used in either the Wide or Narrow Tube phases. Although not all of these techniques were Always Effective in the Narrow Tube phase, this would appear to demonstrate a level of task exploration and potentially innovative tool use within both the Wide and Narrow Tube phases. The use of cloth, fruit stones, and sugarcane as tool materials when such tool use was possible represents spontaneous exploration, not driven by necessity, as the Wide Tube phase could be solved simply by dipping a hand directly into the juice.

The response of Group 4, in contrast with Group 3, bears closer resemblance to studies such as Davis et al. (2016), Manrique et al. (2013), and Lehner et al. (2011), in which some great apes successfully relinquished previously successful techniques in favor of novel solutions to artificial foraging tasks. In contrast to Davis et al. (2016), in the current study effective solutions had to be invented by group members and were not experimentally seeded, and so, although the presentation of the task in a group context may have provided the opportunity to socially learn solutions, chimpanzees were challenged with inventing novel task solutions, like the orangutans studied by Lehner et al. (2011) and the great apes studied by Manrique et al. (2013). Unlike the tasks used by Davis et al. (2016) and Manrique et al. (2013), the current task required the use of tools, which is believed to present a greater challenge in terms of causal cognition (Seed et al., 2009; Völter and Call, 2014). Despite this, like the orangutans given a very similar task by Lehner et al. (2011), chimpanzees in Group 4 not only responded flexibly to the change in task parameters but also invented novel tool-composite techniques that solved the task during the Narrow Tube phases. It is possible that the chimpanzees' experience of the Wide Tube phase provided them with an understanding of the causal relationships and affordances involved in the task, facilitating their innovation in the Narrow Tube phase. Solving a task using a body part, before the task is modified to necessitate tool use, may aid in finding tool use solutions to a problem. Von Bayern et al., 2009 and Jacobson and Hopper (2019) argue that a causal understanding of the task provided in their study allowed chimpanzees and western lowland gorillas (*Gorilla gorilla gorilla*) to respond flexibly to a change in parameters. Although it is possible that, in comparison with tests of flexibility such as multi-access boxes, innovation in the latter stages of the current task was facilitated by the chimpanzees' experience in the Wide Tube phase, this cannot explain the difference in performance seen between Group 3 and Group 4 or the difficulty chimpanzees have had adapting to similar changing foraging tasks (Harrison and Whiten, 2018).

### Tool-composite techniques

The tool-composite techniques observed by Lehner et al. (2011) (described as “Drop-and-Fish” and “Squash-and-Fish”, 2011, pp.450) are argued by the authors to constitute “cumulative technology,” because these techniques were only observed in individuals that had previously performed the component techniques (i.e., individuals that had already used paper as an absorbent material and had used a stick to retrieve debris from within the tube and could then combine these behaviors to form the tool-composite techniques). The authors therefore argue that these behaviors fulfill the criteria to be considered “ratcheted” techniques, as they build upon previous solutions, resulting in more complex techniques that are more widely applicable (as they can be used successfully in both the Wide and Narrow Tube task type). However, because these techniques were elicited by restricting the other solutions available to the orangutans, the extent to which they represent evidence of a capacity for cumulative technology under stable conditions is questionable. The orangutans did not have to overcome satisfaction with their current technique or forgo small rewards in order to invent techniques that would gain even greater rewards, which seems necessary for cumulative culture to emerge in a stable context. In the current study, the potential difference between forced and spontaneous behavioral flexibility can be seen in Group 4's response to the Narrow Tube and Narrow Restricted phases. When individuals had to invent novel techniques and behave flexibly in order to solve the task at all (i.e., in the transition from the Wide to the Narrow Tube phase), they were capable of doing so. However, when the Always Effective technique *stick dip* remained possible in the transition from the Narrow to Narrow Restricted phase, chimpanzees continued to use this technique rather than engaging in exploration of alternative tool-composite techniques for the vast majority of their attempts. This may indicate that chimpanzees do not readily overcome satisfaction with known behaviors to a sufficient extent in order to modify and improve upon them, at least within the relatively short time frame of an experimental task. Such conservatism when known solutions still function has previously been shown in chimpanzees (Marshall-Pescini and Whiten, 2008; van Leeuwen and Call, 2017).

Although it is challenging to track the diffusion of spontaneous innovations in the naturalistic group testing context of the current study, three of the six individuals who used tool-composite techniques in Group 4 did so after having the opportunity to observe others performing a tool-composite technique at the task, suggesting that social learning may have facilitated the spread of these complex behaviors (see Figure S3 for an illustration of the potential chain of diffusion). We cannot, however, rule out the possibility that individuals learned these techniques asocially. Although the tool-composite techniques observed in the current study could be considered, like the tool-composite behaviors observed by Lehner et al. (2011), to be combinations of previous solutions (the combination of dipping an absorbent material by hand, using a stick to dip into the juice, and retrieving an absorbent material using a stick), we did not find that performance of the most complex *stick push and retrieve* techniques was limited to individuals that had first mastered the component behaviors. Jack, the first individual to use such a technique, successfully performed *stick push cloth and retrieve* without first using a stick to simply retrieve material from the tube. Jack had been in proximity to the task during attempts by other individuals using these component behaviors, and so, it is possible that he had gained important information about these simpler tool-composite techniques through observation, which allowed him to then perform the more complex technique without first mastering its component techniques. However, it is also possible that he invented the entire technique rather than socially acquiring the component behaviors. This is an important distinction when considering these results in the context of cumulative culture, for which some authors (e.g., Tennie et al., 2009) specify a criterion that resulting technologies are beyond the capacity of any one individual to invent (although we note that others do not consider this a criterion of cumulative culture, but rather a likely eventual outcome after repeated cycles of cumulation: Mesoudi and Thornton, 2018).

The chimpanzees in both groups at the CWO responded to all phases of the task with tool-use behaviors that broadly correspond to behaviors observed in wild chimpanzees, with the obvious addition of non-natural tool materials such as cloth. Sugarcane fibers were used by chimpanzees at the CWO in a manner similar to wadge-dipping (Boesch, 1991), in that the fibrous inner part of the sugarcane was first chewed to produce a clump of fibers that could then be dipped into the liquid. Wild chimpanzees have also been observed to use probe tools to retrieve water, using either leaves or twigs to dip into water, and a similar probing behavior is used to dip honey from nest cavities (Kummer and Goodall, 1985; Sanz and Morgan, 2007). This is a similar technique to the *stick dip* observed in both groups in the current study and is referred to as “fluid-dip” by Whiten et al. (1999).

Tool-composite techniques were a subject of interest in the current study. These techniques were observed in both groups, although to a greater extent in Group 4 than in Group 3 (both in terms of frequency of use and variety of materials employed). In wild chimpanzees, the tool-composite example most relevant to the current study is that documented by Sugiyama (1997) in which a juvenile female chimpanzee at Bossou was observed using a stick to push a leaf sponge into a tree hollow of water and then to retrieve the leaf sponge again using the stick (note that nutcracking in which the anvil is transported also constitutes a tool-composite behavior, Sakura and Matsuzawa, 1991). This is a very similar instance of tool use to the tool-composites observed in the current study at the CWO, where individuals used stick tools to retrieve absorbent materials from the tube or to push and then retrieve absorbent materials from the tube. According to Whiten et al. (2001), this “sponge push-pull” behavior has also been observed at Tai, Mahale M, and Gombe. The use of tool-composites has only been documented (in wild ape populations) in orangutans (Fox and Bin’Muhammad, 2002) and chimpanzees (Sakura and Matsuzawa, 1991; Sugiyama 1997). To our knowledge, use of tool-composites has not yet been documented in wild New or Old World monkeys, prosimians, or birds (Shumaker et al., 2011). The rarity of tool-composite behavior in wild non-human animals may be due to its complexity, as it requires an animal to take account of the relationships between multiple objects and the target (Sanz and Morgan, 2010), but it may also be due to a limited range of ecological problems that can be solved using tool-composites.

### Scaffolding

Scaffolding provided to Group 3 in the Narrow Scaffolded phase was intended to approximate the physical artifacts produced through tool use that chimpanzees would encounter in the wild, which are suggested to promote the acquisition of new behaviors (Tennie et al., 2009; Fragaszy et al., 2013). However, none of the chimpanzees that encountered the scaffolded solution acquired a novel tool-composite solution as a result. As discussed in Harrison and Whiten (2018), it seems that this physical information alone may not be sufficient to seed novel tool use behaviors in chimpanzees. Along with Gruber et al. (2011), Cardoso and Ottoni (2016), and Harrison and Whiten (2018), the current study forms a limited experimental literature on whether primates are able to acquire novel behaviors simply through the availability of physical artifacts left by others' tool use (in all of these studies, these artifacts are the result of experimental manipulation). None of these studies found that this information was sufficient for a novel behavior to emerge. Enduring physical artifacts aid in chimpanzees' and bearded capuchin monkey's acquisition of skills in the wild (Fragaszy et al., 2013) and are also part of the development of tool use behaviors such as leaf sponging (Sousa et al., 2009). However, they may encounter these artifacts in a broader social context, which could facilitate the acquisition of behaviors in a way the scaffolding manipulation in this study could not. For example, within a social context, it may be possible to observe actions rather than being limited to interaction with artifacts (see Hopper et al., 2015b, for evidence that animate conspecific demonstration facilitates behavior acquisition) or there may be greater motivation to acquire a behavior (see Watson et al., 2018, for evidence captive chimpanzees alter their behavior more readily in group than dyadic contexts). Although some individuals in Group 3 had performed tool-composite techniques prior to the scaffolding, so there was the potential for social observation of this behavior along with interaction with the scaffolding, there were only 12 attempts at this behavior (and only 4 successful attempts), making any social information available very limited. Future research could examine the extent to which a combination of physical scaffolding and social observation may facilitate the acquisition of novel behaviors in controlled conditions, and consider the relative exposure to each type of information that might be necessary.

This study found group differences in chimpanzees' behavioral flexibility in response to a changing artificial foraging task. Chimpanzees in one group were unable to relinquish previously successful techniques when task parameters were altered, and demonstrated only limited behavioral flexibility, whereas another group was able to abandon previously successful techniques in favor of more effective solutions. This group also invented novel, complex, tool-composite behaviors to solve the task when task constraints demanded it and even made limited attempts to modify these complex techniques when materials were restricted. Our finding of group differences in apparent behavioral flexibility as a likely resultant of differential levels of social tolerance at the foraging task may, along with task complexity and differences in experimental paradigms, explain the mixed results in previous studies of chimpanzee behavioral flexibility and highlights the need to test multiple groups and consider intergroup differences when studying animal behavior and cognition.

### Limitations of the study

Low incidence rates of relevant social behaviors at the task precluded detailed statistical exploration of social tolerance at the task, beyond a broad categorization of behaviors as positive or negative indicators of tolerance. However, when considered in the context of previous research demonstrating differences in sociality between the two groups (Cronin et al., 2014a; van Leeuwen et al., 2018), this indicates a potential effect of social tolerance that warrants further investigation.

Our conclusion regarding the link between social tolerance and enhanced performance on a problem-solving task is necessarily limited by the fact that only two groups were tested and compared and only one artificial foraging task was presented. Although our findings provide a robust first step, future research would benefit from including multiple tests of behavioral flexibility at both the group and individual levels and incorporating multiple groups in order to strengthen the conclusions that can be drawn from comparisons between them.

### Resource availability

#### Lead contact

Further information and requests for resources should be directed to and will be fulfilled by the Lead Contact, Andrew Whiten (a.whiten@st-andrews.ac.uk).

#### Materials availability

This study did not generate new unique reagents.

#### Data and code availability

The datasets generated during this study are available at the Open Science Framework [https://osf.io/znhj7/?view_only=0598d06189ac44afb0389f1d0b236181].

## Methods

All methods can be found in the accompanying Transparent methods supplemental file.

## Supplemental information

Document S1. Transparent methods, Figures S1–S3, and Tables S1–S6
