# Supplementary material for: Chimpanzees' behavioral flexibility, social tolerance, and use of tool-composites in a progressively challenging foraging problem
Source: iScience. 2021 Jan 5;24(2):102033. doi: 10.1016/j.isci.2021.102033 (PMC7820130; doi:10.1016/j.isci.2021.102033)
Supplement: Document S1. Transparent methods, Figures S1–S3, and Tables S1–S6 [file mmc1.pdf]

iScience, Volume *24*

Supplemental Information

Chimpanzees' behavioral flexibility, social tolerance, and use of tool-composites in a progressively challenging foraging problem

Rachel A. Harrison, Edwin J.C. van Leeuwen, and Andrew Whiten

Supplemental Information

Supplemental Results

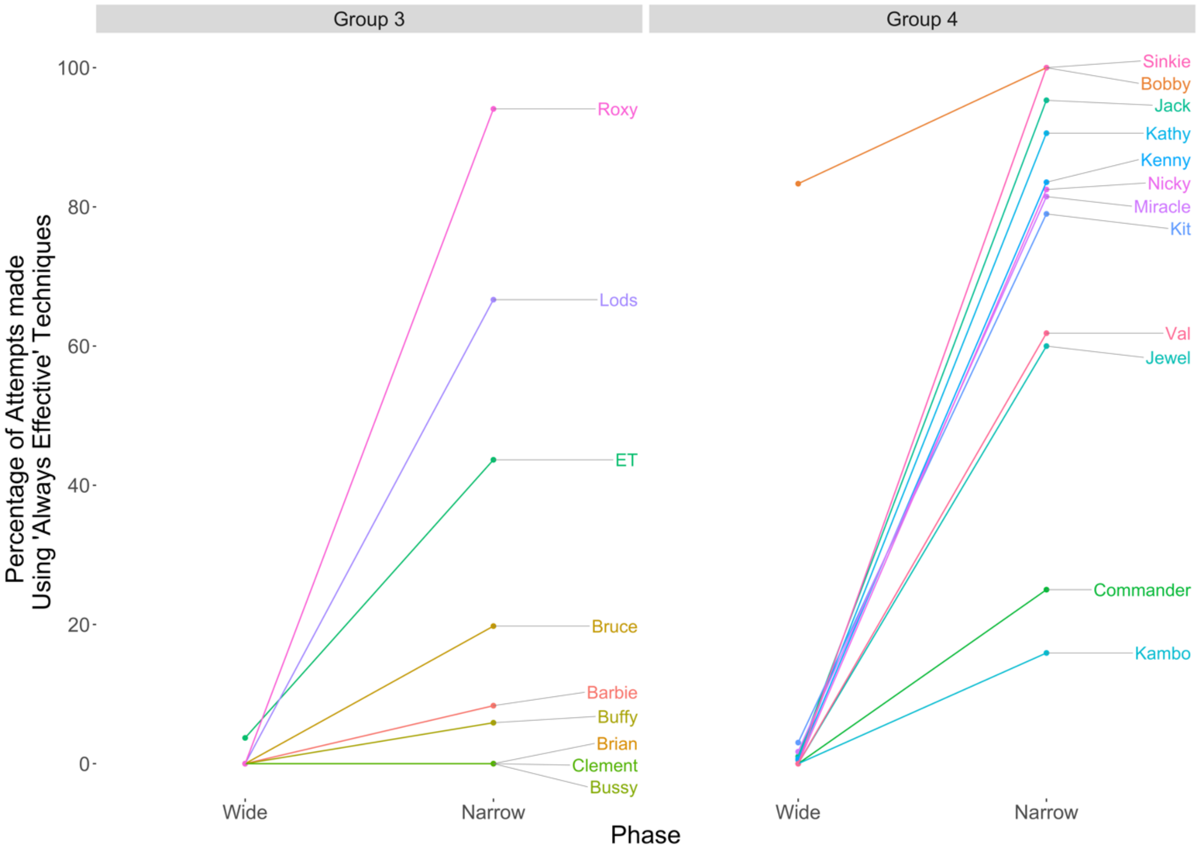

Figure S1: Observed percentages of 'Always Effective' attempts in the 'Wide' and 'Narrow' tube phases, labelled by individual. *Related to Figure 2.*

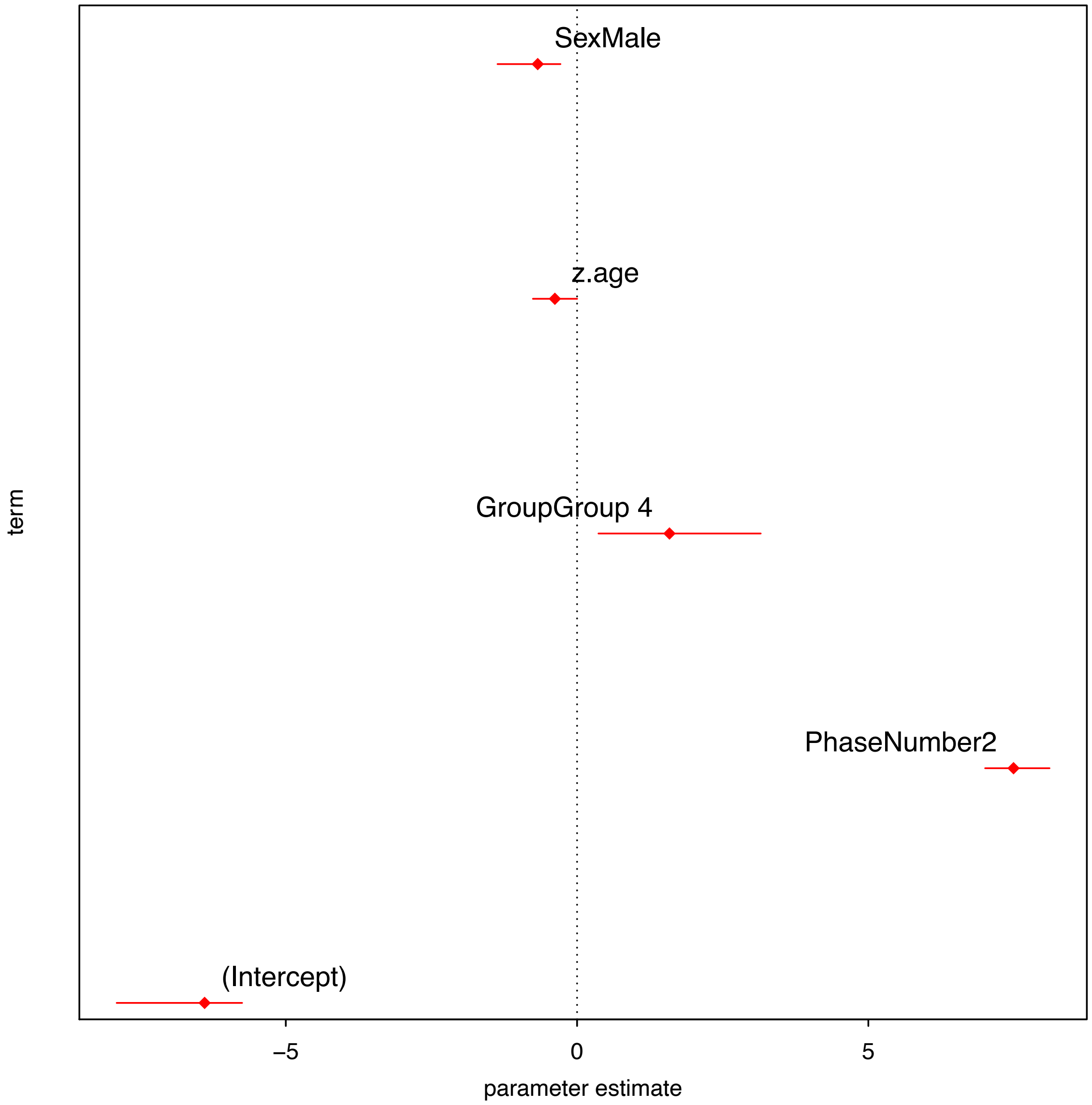

Figure S2: Model stability check. *Related to Table 3.*

Lines indicate the range of parameter estimates based on sequential removal (with replacement) of single subjects and re-running the model (red diamonds represent estimates based on full sample). The obtained estimate-ranges are judged to be reasonably stable, with both "Group" and "PhaseNumber" consistently indicating significant influence on the response variable in the same direction.

Table S1: Top two most frequently attempted techniques by each individual in each phase. Always Effective techniques are shown in bold. *Related to Tables 1 and 2.*

Group	Individual	Wide tube phase		Narrow tube phase		Restricted / Scaffolded phase	
		1st preferred technique (no. of attempts; % of attempts)	2nd preferred technique (no. of attempts; % of attempts)	1st preferred technique (no. of attempts; % of attempts)	2nd preferred technique (no. of attempts; % of attempts)	1st preferred technique (no. of attempts; % of attempts)	2nd preferred technique (no. of attempts; % of attempts)
**Group 3**	Brian	Hand dip (258; 100%)		Hand dip (9; 100%)		Cloth dip (7; 28%)	Hand dip (5; 20%)
	Clement	Hand dip (9; 53%)	Cloth drop (5; 29%)	Hand dip (1; 100%)		Hand dip (1; 100%)	
	Bussy	Hand dip (9; 100%)		Hand dip (2; 100%)		Cloth retrieve (1; 100%)	
	Buffy	Cloth dip (60; 85%)	Hand dip (5; 7%)	Cloth dip (14; 82%)	Hand dip (2; 11%)	**Stick retrieve (6; 54%)**	Cloth dip / Stick dip (2; 18%)
	Roxy	Hand dip (979; 100%)		**Stick dip (281; 92%)**	Hand dip (11; 4%)	Hand dip (2; 67%)	**Stick drop (1; 33%)**
	ET	Hand dip (26; 96%)	**Stick drop (1; 4%)**	Hand dip (37; 52%)	**Stick dip (19; 27%)**	**Stick dip (13; 37%)**	Hand dip (11; 31%)
	Barbie	Cloth drop (63; 53%)	Hand dip (37; 31%)	Hand dip (7; 58%)	Cloth dip (4; 33%)		
	Bruce	Hand dip (664; 92%)	Cloth drop (25; 3%)	Cloth dip (29; 33%)	Hand dip (25; 29%)	Cloth retrieve (6; 43%)	**Stick retrieve (4; 29%)**
	Lods	Hand dip (365; 76%)	Cloth drop (34; 7%)	**Stick dip (5; 56%)**	Hand dip (3; 33%)	**Stick dip (28; 80%)**	**Stick drop (4; 11%)**
**Group 4**	Commander	Hand dip (42; 29%)	Cloth dip (35; 24%)	Paper dip (2; 50%)	Cloth dip / **Stick retrieve** (1; 25%)		

Val	Cloth dip (241; 72%)	Cloth drop (55; 16%)	**Stick dip (52; 54%)**	Cloth dip (30; 31%)	Coconut husk dip / Coconut husk retrieve (1; 50%)	
Nicky	Cloth mouth drop (132; 35%)	Paper dip (51; 14%)	**Stick dip (63; 79%)**	Cloth dip (9; 11%)	**Stick dip (32; 94%)**	**Stick retrieve /** plastic wrapper dip (1; 3%)
Sinkie	Cloth dip (11; 85%)	Cloth drop (2; 15%)	**Stick dip (3; 75%)**	**Stick drop (1; 25%)**	**Stick retrieve (1; 100%)**	
Bobby	**Stick dip (4; 67%)**	Hand dip / **Stick drop** (1; 17%)	**Stick dip (6; 100%)**		**Stick retrieve (1; 100%)**	
Kit	Hand dip (24; 73%)	Cloth dip (4; 12%)	**Stick dip (73; 61%)**	Hand dip (11; 9%)	**Stick dip (3; 100%)**	
Jack	Hand dip (94; 97%)	Cloth dip / Paper retrieve / Stick dip (1; 1%)	**Stick dip (2031; 89%)**	Cloth dip (46; 2%)	**Stick dip (909; 98%)**	**Stick retrieve (5; 0.5%)**
Kathy	Hand dip (138; 97%)	Cloth dip (2; 1%)	**Stick dip (600; 75%)**	**Stick retrieval cloth (40; 5%)**	**Stick dip (20; 80%)**	**Stick retrieval fruit shell (2; 8%)**
Kambo	Cloth drop (64; 45%)	Cloth mouth drop (51; 36%)	**Cloth dip (17; 39%)**	Cloth retrieve (16; 36%)		
Miracle	Cloth mouth drop (20; 34%)	Hand dip (17; 29%)	**Stick dip (15; 56%)**	Cloth dip (5; 19%)	**Stick dip (54; 87%)**	**Stick retrieve (4; 6%)**
Kenny	Hand dip (166; 86%)	Cloth drop (19; 10%)	**Stick dip (127; 80%)**	Hand dip (18; 11%)	**Stick dip (78; 99%)**	Coconut husk dip (1; 1%)
Jewel	Hand dip (95; 100%)		**Stick retrieve (5; 50%)**	Hand dip (4; 40%)		

Table S2: Tool-composite attempts in the 'Narrow Restricted' phase made using novel tool materials. *Related to Tables 1 and 2.*

Individual	Attempted technique (number of attempts)	Latency (from beginning of 'Narrow Restricted' phase) hh:mm:ss	Success
Jack	Straw push and retrieve (x1)	00:03:45	Yes
Jack	Plastic wrapper push and retrieve (x2)	00:37:22	No
Jack	Plastic wrapper retrieve (x3)	00:43:07	Yes (once)
Miracle	Straw push and retrieve (x1)	04:43:22	No
Kathy	Fruit shell retrieve (x2)	04:59:49	No

Table S3: Chimpanzees' interactions with the scaffolded solution of a stick and piece of cloth pre-inserted into the tube. *Related to Tables 1 and 2.*

Individual	Individual's exposure to scaffolding	Interaction with scaffolding
Brian	First exposure	Pulls at end of cloth, which is protruding from top of tube. Does not interact with stick.
	Second exposure	Removes stick from tube (without cloth) and gets juice from it (*stick retrieve* technique).
	Third exposure	Removes stick from tube (without cloth). Unclear if he gets juice from it (*stick retrieve* technique).
	Fourth exposure	Pulls on stick to retrieve stick and cloth from tube, gets juice from both and then discards.
Bruce	First exposure	Pulls at end of cloth, which is protruding from top of tube. Then pulls at stick but does not remove from tube. Finally pulls cloth off stick and discards it (without getting any juice).
ET	First exposure	Manipulates stick inside tube but does not retrieve stick or cloth. Eventually removes stick from tube (without cloth) and gets juice from it (*stick retrieve* technique).
	Second exposure	Pulls on end of cloth to retrieve stick and cloth from tube, gets juice from both, then spends time detaching cloth from stick.

Table S4: A summary of tool-use in a potential foraging context observed during eight hours of observational data collection in each group. *Related to Tables 1 and 2.*

Tool-use in display or self-grooming is not included.

Group	Tool material	Action	Instances observed	Individuals performing
Group 3	Stick	Scratching ground	1	Bruce
	Stick	Reaching under enclosure fence	2	Bruce, ET
Group 4	Stick	Scratching ground	11	Bobby (10 instances), Kenny (1 instance)
	Stick	Reaching under enclosure fence	2	Kenny
	Stick	Probing faeces	1	Nicky

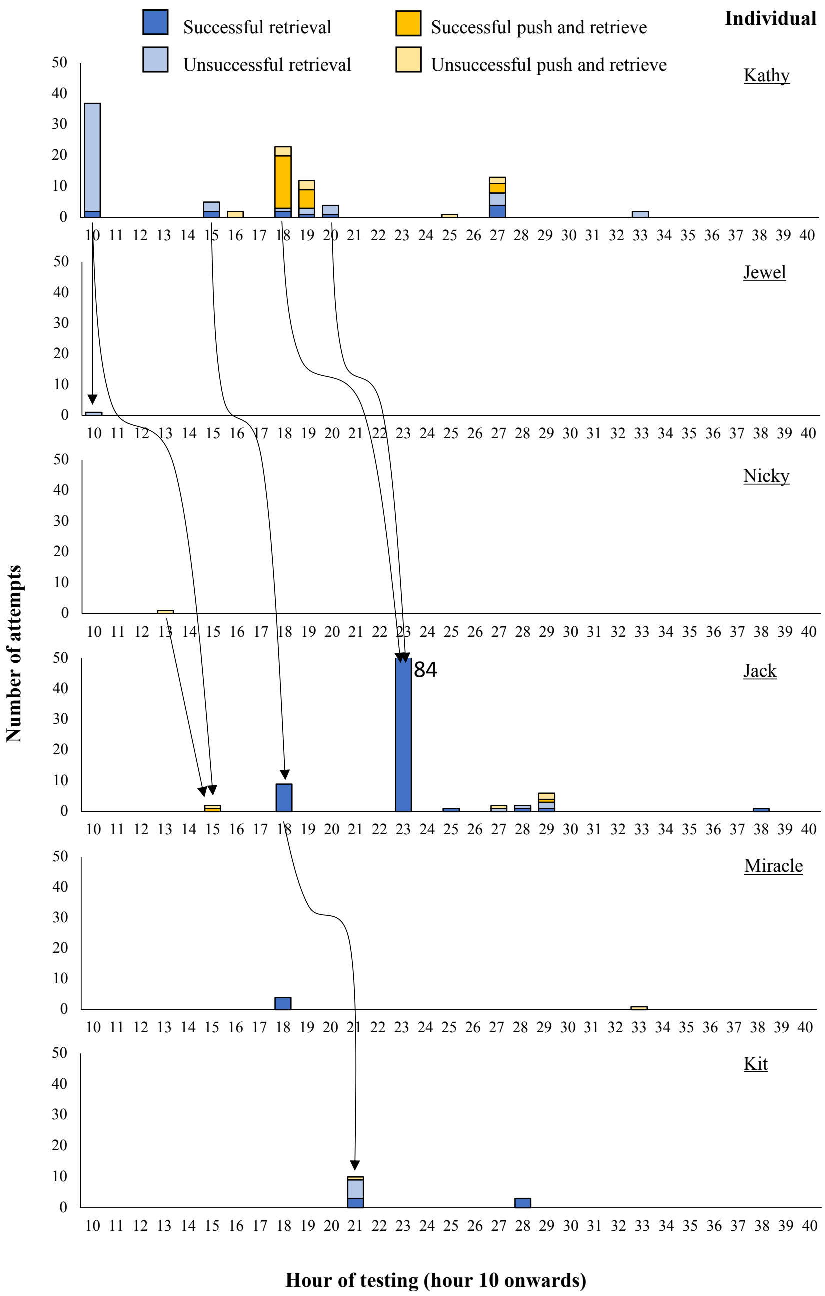

Figure S3: The emergence of tool composite techniques amongst six individuals in Group 4 in the Narrow and Narrow Restricted phases. *Related to Tables 1 and 2.*

Successful and unsuccessful attempts at 'retrieval' and 'push and retrieve' techniques are shown across the 30 hours of testing following the emergence of such techniques. Potential observations by one individual of another attempting tool-composite techniques (instances in which the observer was within 5 metres of the task during an attempt) are represented by arrows connecting the observed session with the next tool composite technique attempted by the observer. All potential observations are represented, whether or not they preceded the observer's first tool-composite attempt. Sessions in which the number of tool composite attempts exceeded 50 are labelled with the number of attempts.

Transparent Methods

Ethical statement

Ethical approval for this study was received from the University of St Andrews School of Psychology and Neuroscience Ethics Committee. In addition, the study was approved by the Chimfunshi Research Advisory Board. The research was carried out in accordance with the guidelines of the Association for the Study of Animal Behaviour.

Subjects and study site

Subjects were 22 chimpanzees housed in two separate groups ('Group 3' and 'Group 4': see Table 1 for details) at the Chimfunshi Wildlife Orphanage (CWO) in northern Zambia. The groups are housed in 47 acre (Group 3) and 62 acre (Group 4) forested enclosures. Chimpanzees in Groups 3 and 4 are able to hear the other group, and there is a stretch of fence line (approx. 3m) where it is possible for chimpanzees from the two groups to see one another (note that the artificial foraging task could not be observed by members of the other group). Chimpanzees at CWO sleep outside overnight, and come indoors for feeding once a day between 11:30 and 13:30. While CWO houses multiple groups with which it is possible to conduct research, the two groups in the current paper were selected due to their size (Group 3: N=10, with N=9 participating in the current study, Group 4: N=12), as it was judged that all group members would be able to have an opportunity to interact with the task, while other groups at CWO are much larger, increasing the likelihood of the task being monopolised by only a subset of the group. In addition, at the time of the study, the two groups in the current paper were the most consistent in all group members entering the indoor enclosure for feeding once a day, providing regular opportunities for keepers or RH to safely

enter their outdoor enclosure to fit and adjust the task, and retrieve tool materials when necessary.

Behavioural flexibility task - Apparatus

Echoing the earlier studies of Lehner et al. (2011) and Harrison and Whiten (2018) two polycarbonate tubes provided the two stages of the task. The first ('wide') tube measured 30cm tall with a 10cm diameter, and the second ('narrow') tube measured 30cm tall with a 5cm diameter. These tubes were bolted to a mesh door attached to the chimpanzees' indoor facility, facing out into their enclosure, and could be filled with diluted juice reward and emptied by the experimenter through the mesh. The task was presented in part of the enclosure where it was not possible for chimpanzees from either group to observe individuals from the other group.

Alongside the tubes, chimpanzees were provided with a selection of tool materials comprising plain sticks, straw, leafy sticks (most frequently bamboo which grows throughout the sanctuary), strips of cloth, and strips of paper. These tool materials were dropped into the enclosure beside the apparatus at the start of each session. Sufficient amounts of each material were provided at the beginning of each session for all group members to use them if they chose; however, it was not possible to prevent chimpanzees from removing materials from the immediate testing area, though these remained within the chimpanzees' enclosure. As fresh materials were provided at the start of each session, materials could also accumulate in the testing area across sessions. Chimpanzees could also use any materials found within their enclosure to attempt the task, and could also use food provided as part of their diet to attempt the task. The use of sugar cane as an absorbent material (specifically, the internal fibrous part of the cane, which could be chewed and was absorbent) occurred in both groups. This sugar cane was provided to chimpanzees by CWO staff as part of their regular diet.

Table S5. Transparent Methods: *Demographic and Rearing Information of the Chimpanzees.*

Group 3
(N = 10)

Individual	Sex	Year of birth (age at time of testing)	Age category*	Mother (if present in group)	Origin	Rearing
Brian	M	1994 (21)	Adult		Wild-born	Hand-raised
Clement	M	1993 (22)	Adult		Wild-born	Hand-raised
Bussy	M	2004 (11)	Subadult	Barbie	Captive-born	Mother (in group)
Buffy	F	1985 (30)	Adult		Wild-born	Hand-raised
Roxy	F	1995 (20)	Adult		Wild-born	Hand-raised
ET	F	1995 (20)	Adult		Wild-born	Hand-raised
Barbie	F	1995 (20)	Adult		Wild-born	Hand-raised
Bruce	M	2009 (5)	Juvenile	Barbie	Captive-born	Mother (in group)
Lods	F	2010 (5)	Juvenile	Mother deceased	Captive-born	Mother (in group)
Brent	F	2014 (1)	Infant	Barbie	Captive-born	Mother (in group)

*Age categories following Reynolds (2005), with behavioural characteristics considered in addition to age in years. Note that infant Brent was too young to participate in the current study.

Group 4
(N = 12)

Individual	Sex	Year of birth (age at time of testing)	Age category*	Mother (if present in group)	Origin	Rearing
Commander	M	2001 (14)	Adult		Wild-born	Hand-raised
Val	M	2000 (15)	Adult		Wild-born	Hand-raised
Nicky	M	1991 (24)	Adult		Wild-born	Hand-raised
Sinkie	M	1994 (21)	Adult		Wild-born	Hand-raised
Bobby	M	1993 (22)	Adult		Wild-born	Hand-raised
Kit	M	2005 (10)	Subadult	Kambo	Captive-born	Mother (in group)
Jack	M	2008 (7)	Juvenile	Mother deceased	Captive-born	Mother (in group)
Kathy	F	1999 (16)	Adult		Wild-born	Hand-raised
Kambo	F	1996 (19)	Adult		Wild-born	Hand-raised
Miracle	F	2000 (15)	Adult		Captive-born	Hand-raised
Kenny	M	2011 (4)	Infant	Kambo	Captive-born	Mother (in group)
Jewel	M	2013 (2)	Infant	Mother deceased Kathy (adoptive mother)	Captive-born	Hand-raised

*Age categories following Reynolds (2005), with behavioural characteristics considered in addition to age in years

Behavioural flexibility task - Procedure

In the first stage of the study ('Wide Tube' phase), chimpanzees were provided with the wide tube, filled with diluted juice to a depth of around 7cm. This was presented alongside the aforementioned selection of tool materials. The apparatus remained fitted within the chimpanzees' enclosure 24 hours a day during the testing period, but was filled with juice reward for only 10 hours of testing in total. Testing sessions lasted between one and three hours, and up to two sessions were held per day: in the morning prior to the chimpanzees' indoor feeding at 11:30 and in the afternoon post-feeding after 13:30, when the chimpanzees were released back into their outdoor enclosure. Chimpanzees had access to the task within their enclosure as a group. The tube was refilled with reward by the experimenter during testing when the chimpanzees had emptied it, and was emptied of reward by the experimenter at the end of each testing session.

In the second stage ('Narrow Tube' phase), only the narrow tube was presented, alongside the same selection of tool materials. The tube was filled with reward for 20 hours of testing, again in sessions lasting between one and three hours. The narrow tube prevented chimpanzees from inserting their hands into the tube in order to solve the task.

The third stage of testing differed for Groups 3 and 4, in response to the chimpanzees' behaviour in the previous two stages. Group 4 had responded to the 'Narrow Tube' phase with the use of innovative, tool-composite techniques, and so, as in Lehner et al. (2011), we removed tool materials which were being used successfully in order to test for further potential flexibility and technique modification. Group 4 was therefore provided with the narrow tube for a further 10 hours, but was no longer provided with cloth or sugar cane (part of the chimpanzees' diet provided occasionally by their keepers), two absorbent materials

used successfully by the group as components of tool-composite techniques in the previous phase (this intervention formed the 'Narrow Restricted' phase).

Group 3 had limited success in the 'Narrow tube' phase, and did not show extensive use of tool-composite techniques, so we provided scaffolding towards such a technique that had emerged in Group 4 in a manner similar to a previous study with zoo-housed chimpanzees (Harrison & Whiten, 2018). Group 3 was therefore provided with the narrow tube with a plain stick with a piece of cloth tied to the end inserted into the tube prior to the start of each session (this intervention formed the 'Narrow Scaffolded' phase).

<u>Behavioural flexibility task - data collection and coding</u>

Chimpanzees' responses to the task were video recorded while the experimenter (RH) narrated chimpanzees' actions as well as the identity and presence of other individuals in the task area. The video and narration were later coded together by RH. The identity, tool material choice, action and success of individuals attempting the task were coded for each attempt. The presence of other individuals at the task or in the task area, and their distance from the task, was also coded from RH's narration for each attempt.

Tool material and action were the two variables which indicated what technique an individual had used, and thus formed the basis for the analysis of behavioural flexibility. A second coder who was blind to the study hypotheses coded 632 randomly selected attempts (approximately 5% of all attempts) for tool material and action. Inter-observer reliability was calculated using an unweighted Cohen's Kappa, revealing good agreement for both variables (tool material K = 0.97, action K = 0.81).

Techniques were classified as 'Always Effective' or 'Initially Effective' based upon their efficacy across the 'Wide' and 'Narrow Tube' phases. Techniques which

involved inserting a hand into the tube were classified as 'Initially Effective', as while they could be used in the 'Wide Tube' phase, the width of the tube in the 'Narrow Tube' phases prevented their use.

Data analysis

Data were analysed in R (version 3.2.2, R Core Team, 2013) and RStudio (version 0.99.893, RStudio Team, 2015), using a generalised linear mixed model with a Laplace approximation (GLMM), via the function glmer in the R package lme4 (Bates et al., 2012). The impact of predictor variables upon the number of 'Always Effective' vs 'Initially Effective' technique attempts in the 'Wide Tube' and 'Narrow Tube' phases was assessed. The full model contained fixed effects for all variables of interest: the effect of Phase ('Wide Tube' vs. 'Narrow Tube'), Group (Group 3 vs Group 4), Sex (Male vs Female) and Age (scaled continuous variable) along with a random intercept and slope by Phase for each Individual (N=21), and was compared with a null model (containing only the random intercept and slope by Phase for each Individual). Both models were fitted using a binomial error structure due to the binary nature of the response variable (Effectiveness, with 'Initially Effective' attempts as the reference level) and a logit link function. The full model was assessed for stability by excluding individuals one at a time from the model and comparing the resulting parameter estimates with those obtained from using the entire data set. No influential cases were found to exist. See Figure S2 for illustration of model stability. Variance inflation factors were derived using the function vif in the R package car (Fox & Weisberg, 2019) and indicated no substantial collinearity between predictor variables (maximum VIF: 1.33).

Data were plotted using the R package ggplot2 (Wickham, 2016) and cowplot (Wilke, 2019). Model predictions were generated using the R package ggeffects (Lüdecke, 2018).

Social tolerance – Coding social behaviours during the behavioural flexibility task

All video records from the task were coded by RH with a specific focus upon social interactions relevant to social tolerance. Behaviours assumed to be 'positive' and 'negative' indicators of social tolerance were coded for. The behaviours coded for, along with their definitions, are provided in Table 2. A short review explaining the relevance of each behaviour to social tolerance is provided below. All individuals visible on these video records were included in the coding (i.e. interactions between individuals were coded whenever they were visible, not only when one individual was active at the task).

For each of these social behaviours, the time of occurrence, identity of the individual active at the task (or occupying the space directly in front of the task), the identity of the second individual involved, the type of behaviour, the direction of the behaviour (eg. individual A displaces individual B; individual A scrounges from individual B), and the identity of the individual active at the task following the behaviour were recorded. In addition, notes described pertinent details of the behaviour (e.g. for episodes of peering, the technique being observed by the peering individual was noted; for tool transfers the transfer was described in detail).

In addition, RH coded the video records to measure the amount of time each group spent at the task. This was a binary measure, recorded whenever at least one individual was present at the task (with 'at the task' meaning an individual was either sat at the mesh to which the task was attached, or visible sitting on the ground directly below the task). The measure of how long each group spent physically present at the task provides some context for the frequency of social behaviours observed, as such behaviours could only be recorded from this video record during times at which individuals were present at the task.

Aggression at the task

Aggression between individuals is likely to impede innovation (if the individual acting on the task is aggressively displaced by another chimpanzee), and may also impede the possibility of social learning (if the individual acting upon the task threatens or aggresses potential observers in order to maintain control of the task) (van Schaik, Deaner & Merrill, 1999). An artificial foraging task presents a monopolizable resource, potentially increasing the chance of aggressive conflict between individuals (Wittig & Boesch, 2003). Willingness to co-feed from a monopolizable food resource has been used as a measure of social tolerance, with bonobos (considered a more socially tolerant species) being more likely to co-feed than chimpanzees, and showing no aggression during co-feeding, while aggressive interactions were observed (though rarely) in chimpanzees (Hare et al., 2007; but see Jaeggi et al., 2010; Cronin, de Groot & Stevens, 2015). The ability to access an artificial foraging task with infrequent or limited aggression may lead to greater success (as individuals can perform the task uninterrupted), and may also indicate a level of social tolerance through the ability to co-feed at a monopolizable resource.

Displacement

Displacements, (also termed 'approach-retreat' or supplant interactions), are often used as a means of assessing dominance hierarchies in non-human animals (Silk et al., 2010; Sicotte, 2002; Alados & Escos, 1992; Boyd & Silk, 1983; Seyfarth, 1976; Rowell, 1974), with higher-ranking individuals more likely to succeed in displacing lower-ranking individuals. While it is the direction, symmetry, and response to displacements, rather than their frequency, that is often referred to in studies of social tolerance (e.g. Sapolsky & Share, 2004), van Schaik (2003) predicts that individuals in socially intolerant groups will be more likely to be displaced from a foraging bout, suppressing their opportunity to innovate. Similarly, Horner (2010) suggests that social tolerance facilitated chimpanzees' close observation of

demonstrators and subsequent acquisition of a novel tool-use behaviour (reported in Whiten, Horner & de Waal, 2005), as tolerance allowed observers to approach and observe without displacing the demonstrator.

Concurrent action

If individuals are sufficiently socially tolerant to share close proximity at a foraging site, the opportunity arises for concurrent action (defined in our study as *two or more individuals acting upon the task at once*, i.e. making simultaneous but separate attempts, or rapidly alternating attempts). This was possible due to the group testing situation, and due to the nature of the task, which was monopolizable but also amenable to concurrent action if individuals positioned themselves on either side of the tube. In a study conducted with the same groups of chimpanzees at CWO, Cronin et al. (2014) presented dyads of group-members with a task in which a tray containing food rewards hung within a mesh tower. By sitting on top of the tower and pulling on two chains, chimpanzees could lift the tray towards themselves and retrieve the reward. In order to have the opportunity to solve the task collaboratively, individuals would have to both occupy the $1m^2$ panel on top of the tower, and their willingness to do this provides some idea of their ability to concurrently attempt to gain a food reward while in close spatial proximity to one another. Cronin et al. (2014b) found that individuals simultaneously occupied the tower for an average of 11.2% of sessions (or, 1.2 minutes of each 10-minute session), and time spent together on the tower was positively correlated with a dyad's proximity-based social network association index. Cronin et al. (2014b) also found that monopolization of the task was common, with 80% of sessions being entirely monopolized by one partner in a dyad. These results indicate that concurrent action at an artificial foraging task may be relatively rare, but the correlation between a dyad's time spent together on the tower and association index based on free-roaming daily activities implies that a

certain level of inter-individual tolerance is required in order for this to occur, which may make concurrent action a useful indicator of social tolerance.

Coaction

Coaction has been observed in experimental studies of chimpanzee social learning (Horner, Whiten, Flynn & de Waal, 2006; Horner, 2010), and in wild chimpanzees (McGrew, 1977; Sanz & Morgan, 2013) and captive capuchins (Westergaard & Fragaszy, 1987), and describes the situation in which an observer touches the tool or hand of an individual performing a behaviour (Visalberghi & Fragaszy, 1990). Fragaszy and Visalberghi (1990) suggest that coaction requires a high level of social tolerance, and it is thought to facilitate social learning.

Peering

For some primate species, peering (attentive, close-range observation) has been argued to be an important means of acquiring information socially, with wild infant orangutans peering more at their mother when she fed on less-familiar foods which required a greater complexity of processing (Schuppli et al., 2016). Infants then spent more time exploring foods that they had peered at their mother eating. Schuppli et al. (2016) also found that peering decreased as young orangutans became more competent (and so, required less social information). Along with a general reduction in peering behaviour, as juveniles aged, they began to direct a greater proportion of their peering towards individuals other than their mother, allowing them to acquire information beyond that which observing their mother could provide (Schuppli et al., 2016). Yamanashi et al. (2016) found that providing an artificial foraging task to captive chimpanzees elicited peering behaviour, particularly in an infant, who, like the wild orangutans studied by Schuppli et al. (2016), showed selectivity in his peering, tending to peer more at adults following his failed attempts than following successful attempts. Peering therefore appears to

be a means, at least in part, of acquiring social information, and so may have allowed individuals to acquire new solutions to the task, improving performance. Peering may also be an indicator of social tolerance, as in order to peer closely at another's actions, individuals must be in close proximity at the task.

Tolerated scrounging

McGrew and Feistner (1992) suggested that tolerated scrounging (the theft of food with little or no resistance from the owner) is the most common form of food sharing in chimpanzees, and primarily occurs between mother and offspring. In the wild, infant chimpanzees scrounge nut kernels, primarily from their mothers (Inoue-Nakamura & Matsuzawa, 1997). Scrounging has also been argued to facilitate social learning (Caldwell & Whiten, 2003), with common marmosets that had the opportunity to scrounge from a demonstrator during an artificial foraging task more likely to acquire the demonstrated behaviour than those that were only able to observe and not scrounge. We interpret tolerated scrounging as a positive indicator of social tolerance, as it indicates individuals were in close proximity during a foraging bout, and demonstrates tolerance on the part of the individual that has produced the resource being scrounged (at least when this individual is the more dominant of the two, as in mother-offspring scrounging). It should be noted that other authors (Horner, 2010) have suggested that 'excessive' scrounging might discourage proficient individuals from performing a behaviour, and argue that social tolerance might be indexed by limited scrounging.

Tool transfers

Tool transfer is defined by Pruetz and Lindshield (2012) as "the exchange of a resource between one dyad within an uninterrupted feeding or foraging bout". This definition does not require the transfer to be voluntary. Theft, passive and active transfers were all considered examples of tool transfer by Pruetz and Lindshield (2012), and transfers were further categorised by the extent to which

they were actively initiated by the possessor of a tool (following Boesch and Boesch, 1989). Musgrave et al. (2020) split tool transfers into two types – requested and unrequested, and then categorise them by the possessor's behaviour, ranging from prosocial 'requested active' or 'unrequested proactive' transfers, to 'requested passive' or 'unrequested tolerated taking', to 'requested hesitant' or 'unrequested stealing'.

Pruetz and Lindshield (2012) document ten cases of stick tool transfers amongst chimpanzees at Fongoli, Senegal. The authors suggest that there is a high incidence of both tool and food transfers at Fongoli, relative to other sites, and that this fits with expectations for a cohesive community, in which the average party size is more than 40% of the community (Pruetz & Bertolani, 2009). Tool transfers have also been documented in the Goualougo Triangle, Republic of Congo (Musgrave et al., 2016), primarily occurring between adult females and their immature offspring. The authors argue that these transfers constitute teaching, as the tool donors experienced a reduction in feeding events following the transfer, while recipients experienced an increase in feeding events following the transfer. A later study (Musgrave et al., 2020) compared transfer rates at Goualougo with Gombe, finding that transfers occur more frequently at Goualougo, and thus may be critical in supporting the use of complex tool sets at this site. Tool transfers may therefore be considered, along with peering, as a potential source of social information during a tool-based foraging bout. As Pruetz and Lindshield (2012) argue, tool transfers also indicate social tolerance between the donor and the recipient, and this may be why the majority of transfers observed by Musgrave et al. (2016; 2020) were between mother and offspring.

Table S6. Transparent Methods: *Social behaviours recorded at the task, with the definition followed during video coding.*

Behaviour	Coding definition
Aggression	Agonistic behaviour directed at another individual. Included physical contact aggression (eg. biting or hitting) and displays or threats if clearly directed toward another individual.
Displacement	An individual retreats spatially within 5 seconds of another individual approaching.
Co-action	An individual touches the acting individual's hand or part of a tool during use.
Concurrent action	Two or more individuals act upon the task at once (ie. making simultaneous but separate attempts, or rapidly alternating attempts)
Peering	An individual observes another for at least 5 seconds from a distance of less than 1 metre. The head movements of the peering individual follow the demonstrator's actions.
Scrounging	An individual gets juice reward as a result of another's action at the task (ie. the scrounger licks juice from the actor's tool item or hand after the actor has attempted the task)<br><br>In cases where juice was retrieved from a tool, this is distinct from tool transfer in that during scrounging, the tool remains in the possession of the original owner.<br><br>Instances in which absorbent materials were discarded by their owner and taken by an observer are categorised as scrounging.
Tool transfer	The exchange of a tool between two individuals. This included transfers in which one individual took a tool item out of the hands of another chimpanzee, and in which transfer occurred mid-attempt (individual A inserts item into tube, individual B immediately takes over attempt with A's inserted material). Instances in which an individual took an absorbent material that the owner had discarded are classed as scrounging.

Supplementary descriptive results – social tolerance. *Related to Table 4.*

Aggression at the task

In both groups, aggression was a relatively unusual social interaction at the task in comparison to the other social behaviours recorded, and only nine instances of aggression were recorded across both groups. In all cases of aggression, juveniles and infants were the recipients of aggression. Males were the aggressors in eight of the nine instances. In five cases, the aggressor was not the individual active at the task prior to the aggression, and in four cases, the aggressor was the individual already active at the task, suggesting that aggression was used both in attempting to retain control of the task, and in attempting to take control of the task.

Displacement

Displacement was more frequent in Group 3 than Group 4, with displacements occurring more than once per hour (1.71 occurrences per hour) in Group 3, and nearly once an hour (0.95 occurrences per hour) in Group 4. Combining data from both Group 3 and Group 4, juveniles (53%, 22/41 occurrences), adult females (19.5%, 8/41 occurrences), and infants (14.6%, 6/41 occurrences) were the classes most frequently displaced from the task. Both adult males and adult females were observed displacing others, and adults were the most frequent initiators of displacement (39%, 16/41 adult male occurrences, 39%, 16/41 adult female occurrences).

Concurrent action

Concurrent action was observed in both groups, but was observed more frequently in Group 4 than Group 3 (occurring 1.05 times per hour in Group 4, and 0.41 times per hour in Group 3). Four of five instances of concurrent action in Group 3 were between a juvenile male and an unrelated adult female, with the fifth instance

being between the juvenile male and his mother. These instances occurred during the 'Wide Tube' phase, and in all cases the individuals alternated hand or cloth dipping attempts. In Group 4 the majority of instances of concurrent action included a juvenile or infant as one of the partners (86.4%, 19/22 occurrences), but only six of 22 instances (27%) were a mother-offspring partnership.

<u>Coaction</u>

Coaction was observed more frequently in Group 4 than in Group 3. Only two instances were observed in Group 3, both between an adult female and her offspring. In both cases, the female's infant and juvenile sons held either her hand or the cloth while she engaged in cloth dipping at the task. In Group 4, the majority of instances of coaction were between family members. In the majority of cases (84.6%, 11/13), the co-actor (the individual observing an attempt at the task) was an infant, and this meant that in the majority of cases the individual observing was younger than the individual acting on the task. In the majority of instances of coaction in Group 4, the observing individual touched or held a stick tool as the active individual attempted the task, with only one instance of coaction involving any other tool material.

<u>Peering</u>

Instances of peering were observed at a similar rate in both Group 3 and Group 4. In Group 3, only juveniles and infants were observed peering. These individuals most frequently peered at adult females (70.6%, 12/17 instances), but in only four of 17 instances (23.5%) was this peering towards family members. In Group 4, 20 of 28 instances (71.4%) of peering were by juveniles or infants, and as in Group 3, peering occurred towards both family and non-family members (25%, 7/28 instances of offspring towards mother, 10.7%, 3/28 instances between siblings, 64.3%, 18/28 instances between unrelated individuals).

Tolerated scrounging

Scrounging (in the current study: instances in which an individual obtained juice as a result of another's action at the task, either by licking the other's hand or tool, or by taking discarded absorbent materials) occurred more frequently in Group 4 than in Group 3 (1.14 times per hour of observation vs 0.08 times per hour of observation). Scrounging was only observed once in Group 3, in an incident in which a juvenile male licked juice from his mother's hand. In Group 4, 24 instances of scrounging were observed. The majority of these (58.3%, 14/24 cases) were instances in which one infant scrounged from his adoptive mother. Twenty-two of the 24 instances (91.6%) of scrounging observed in Group 4 were either juveniles or infants scrounging from older individuals, and in 18 of 24 cases (75%) the older individual was kin (16 mother-offspring, 2 siblings). In only two instances an older individual scrounged from a younger individual.

Tool transfers

In the current study, tool transfers were observed more frequently in Group 4 than Group 3 (0.33 incidents per hour of observation in Group 3 vs. 1.10 incidents per hour of observation in Group 4). Four tool transfers were observed in Group 3, and in three of these, a juvenile male was the recipient. The juvenile male took tools from both an unrelated adult female and his mother. The fourth instance of tool transfer in Group 3 was an adult female taking a stick from a juvenile female prior to beginning an attempt at the task.

Twenty-three tool transfers were observed in Group 4. All of these events involved a non-adult individual (infant, juvenile or subadult) as either owner or recipient of the tool being transferred. Six instances were transfers between mother-offspring pairs, and in all six such cases the infant was the recipient of the tool. In addition, there were four sibling transfers, but in these cases the transfer was not always in

the direction of the younger sibling, with a subadult male taking a tool from his infant brother on one occasion.

Of the 23 tool transfers observed in Group 4, the majority (56.5%, 13/23) were between non-kin. These included four instances of infant males taking tools from unrelated adults (a male and a female). On three occasions, the same unrelated adult female took tools from the two infant males. This contrasts with Musgrave et al.'s (2016; 2020) findings in wild chimpanzees in the Goualougo Triangle, Republic of Congo, that the majority of tool transfers occur between mother-offspring dyads (with only 8 of 65 transfers reported in Musgrave et al., 2016, occurring between peers or from younger to older individuals).

The participation of non-adult individuals in the tool transfers in Group 4, as either owner or recipient of the tool, suggests two types of transfer in this group. The first (34.8%, 8/23 instances) are those in which an older, more dominant individual takes a tool from a younger individual, and the second (65.2%, 15/23 instances) are those in which an older, more dominant individual tolerates the actions of a younger, subordinate individual (analogous to 'tolerated taking' in Musgrave et al.'s, 2020, classification scheme, though note that we did not code for whether a tool transfer was requested by the recipient, which is required to meet Musgrave et al.'s criteria for active transfer of tools).

Supplementary Results*: Technique repertoires. *Related to Tables 1 and 2.

Individuals varied in the number of techniques (as defined in Tables 1 and 2) that they attempted across the three phases of the study. Individual's preferred techniques are shown in Supplementary Table S3. In Group 3, individuals used a mean of 4.11 techniques in the Wide tube phase (sd = 3.62, range = 1 – 11), 4.11 techniques in the Narrow tube phase (sd = 3.66, range = 1 – 12), and 3.88 techniques in the Narrow Scaffolded phase (sd = 2.30, range = 1 – 7). In Group 4, individuals used a mean of 5.17 techniques in the Wide tube phase (sd = 2.72, range = 1 – 11), 7.25 techniques in the Narrow tube phase (sd = 5.63, range = 1 – 20) and 2.89 techniques in the Narrow Restricted phase (sd = 2.09, range = 1 – 7). Mann-Whitney U tests indicated no significant difference in repertoire size between the two groups in either the Wide (Mann–Whitney $U = 37.5$, $n_1 = 9$, $n_2 = 12$, $P = 0.25$ two-tailed) or Narrow (Mann–Whitney $U = 33$, $n_1 = 9$, $n_2 = 12$, $P = 0.14$ two-tailed) tube phases. Narrow Scaffolded and Narrow Restricted phases were not compared as the experimental methodology differed between groups at this stage. A linear model indicated no effect of repertoire size in the Wide tube phase upon an individual's flexibility (the extent to which they increased their use of Always Effective techniques following the introduction of the Narrow tube) ($F(1,19) = 0.52$, $p = 0.48$). The percentage of attempts made using Always Effective techniques during the Wide tube phase also did not predict the extent to which individuals increased their use of Always Effective techniques in the Narrow tube phase ($F(1,19) = 0.66$, $p = 0.42$), indicating that individuals with experience of these techniques did not increase their use of them to a greater extent than those who had never used an Always Effective technique.

Supplemental references

Alados, C. L., & Escós, J. M. (1992). The determinants of social status and the effect of female rank on reproductive success in Dama and Cuvier's gazelles. *Ethology Ecology & Evolution*, *4*(2), 151-164.

Bates, D., Maechler, M., & Bolker, B. (2012). lme4: Linear mixed-effects models using S4 classes. R package version 0.999999-0.

Boesch, C., & Boesch, H. (1989). Hunting behavior of wild chimpanzees in the Tai National Park. *American Journal of Physical Anthropology*, *78*(4), 547-573.

Boyd, R., & Silk, J. B. (1983). A method for assigning cardinal dominance ranks. *Animal Behaviour*, *31*(1), 45-58.

Caldwell, C. A., & Whiten, A. (2003). Scrounging facilitates social learning in common marmosets, Callithrix jacchus. *Animal Behaviour*, *65*(6), 1085-1092.

Cronin, K. A., De Groot, E., & Stevens, J. M. (2015). Bonobos show limited social tolerance in a group setting: A comparison with chimpanzees and a test of the relational model. *Folia Primatologica*, *86*(3), 164-177.

Cronin, K. A., Pieper, B. A., van Leeuwen, E. J., Mundry, R., & Haun, D. B. (2014). Problem solving in the presence of others: how rank and relationship quality impact resource acquisition in chimpanzees (Pan troglodytes). *PloS one*, *9*(4), e93204.

Fox, J., & Weisberg, S. (2019). *An R Companion to Applied Regression*, Third edition. Sage, Thousand Oaks CA.

Fragaszy, D. M., & Visalberghi, E. (1990). Social processes affecting the appearance of innovative behaviors in capuchin monkeys. *Folia Primatologica*, *54*(3-4), 155-165.

Hare, B., Melis, A. P., Woods, V., Hastings, S., & Wrangham, R. (2007). Tolerance allows bonobos to outperform chimpanzees on a cooperative task. *Current Biology*, *17*(7), 619-623.

Harrison, R. A., & Whiten, A. (2018). Chimpanzees (*Pan troglodytes*) display limited behavioural flexibility when faced with a changing foraging task requiring tool use. *PeerJ*, *6*, e4366.

Horner, V. (2010). The cultural mind of chimpanzees: how social tolerance can shape the transmission of culture. In E. V. Lonsdorf, S. R. Ross & T. Matsuzawa (Eds.) *The Mind of the Chimpanzee: Ecological and Experimental Perspectives*, (pp.101-115). Chicago: University of Chicago Press.

Horner, V., Whiten, A., Flynn, E., & de Waal, F. B. (2006). Faithful replication of foraging techniques along cultural transmission chains by chimpanzees and children. *Proceedings of the National Academy of Sciences*, *103*(37), 13878-13883.

Inoue-Nakamura, N., & Matsuzawa, T. (1997). Development of stone tool use by wild chimpanzees (Pan troglodytes). *Journal of Comparative Psychology*, *111*(2), 159.

Jaeggi, A. V., Stevens, J. M., & van Schaik, C. P. (2010). Tolerant food sharing and reciprocity is precluded by despotism among bonobos but not chimpanzees. *American Journal of Physical Anthropology*, *143*(1), 41-51.

Lehner, S. R., Burkart, J. M., & van Schaik, C. P. (2011). Can captive orangutans (*Pongo pygmaeus abelii*) be coaxed into cumulative build-up of techniques?. *Journal of Comparative Psychology*, *125*(4), 446.

Lüdecke D (2018). *ggeffects: Tidy Data Frames of Marginal Effects from Regression Models.* Journal of Open Source Software, 3(26), 772.

McGrew, W. C. (1977). Socialization and object manipulation of wild chimpanzees. In F.E. Poirer, S. Chevalier-Skolnikoff (Eds.), *Primate Bio-social Development: Biological, Social and Ecological Determinants*, (pp. 261-288).

McGrew, W. C., & Feistner, A. T. (1992). Two nonhuman primate models for the evolution of human food sharing: chimpanzees and callitrichids. In J. H. Barkow, L. Cosmides, J. Tooby (Eds.) *The Adapted Mind: Evolutionary Psychology and the Generation of Culture*, (pp. 229-243). Oxford, UK: Oxford University Press

Musgrave, S., Lonsdorf, E., Morgan, D., Prestipino, M., Bernstein-Kurtycz, L., Mundry, R., & Sanz, C. (2020). Teaching varies with task complexity in wild chimpanzees. *Proceedings of the National Academy of Sciences*, *117*(2), 969-976.

Musgrave, S., Morgan, D., Lonsdorf, E., Mundry, R., & Sanz, C. (2016). Tool transfers are a form of teaching among chimpanzees. *Scientific Reports*, *6*, 34783.

Pruetz, J. D., & Bertolani, P. (2009). Chimpanzee (Pan troglodytes verus) behavioral responses to stresses associated with living in a savanna-mosaic environment: implications for hominin adaptations to open habitats. *PaleoAnthropology*, 252.

Pruetz, J. D., & Lindshield, S. (2012). Plant-food and tool transfer among savanna chimpanzees at Fongoli, Senegal. *Primates*, *53*(2), 133-145

R Core Team (2013). R: A language and environment for statistical computing. R Foundation for Statistical Computing, Vienna, Austria. URL http://www.R-project.org/.

Reynolds, V. (2005). *The chimpanzees of the Budongo forest: Ecology, behaviour and conservation*. OUP, Oxford.

Rowell, T. E. (1974). The concept of social dominance. *Behavioral Biology*, *11*(2), 131-154.

RStudio Team. (2015). RStudio: integrated development for R. RStudio, Inc., Boston, MA URL http://www. rstudio.com

Sanz, C. M., & Morgan, D. B. (2013). Ecological and social correlates of chimpanzee tool use. *Philosophical Transactions of the Royal Society of London B: Biological Sciences*, *368*(1630), 20120416.

Sapolsky, R. M., & Share, L. J. (2004). A pacific culture among wild baboons: its emergence and transmission. *PLoS biology*, *2*(4), e106.

Schuppli, C., Meulman, E. J., Forss, S. I., Aprilinayati, F., van Noordwijk, M. A., & van Schaik, C. P. (2016). Observational social learning and socially induced practice of routine skills in immature wild orang-utans. *Animal Behaviour*, *119*, 87-98.

Seyfarth, R. M. (1976). Social relationships among adult female baboons. *Animal Behaviour*, *24*(4), 917-938.

Sicotte, P. (2002). The function of male aggressive displays towards females in mountain gorillas. *Primates*, *43*(4), 277-289.

Silk, J. B., Beehner, J. C., Bergman, T. J., Crockford, C., Engh, A. L., Moscovice, L. R., Wittig, R. M., Seyfarth, R. M. & Cheney, D. L. (2010). Strong and consistent social bonds enhance the longevity of female baboons. *Current Biology*, *20*(15), 1359-1361.

van Schaik, C. P. (2003). Local traditions in orangutans and chimpanzees: Social learning and social tolerance. In: D. M. Fragaszy, S. Perry (Eds.) *The Biology of Traditions* (pp.297 – 328). Cambridge, UK: Cambridge University Press.

van Schaik, C. P., Deaner, R. O., & Merrill, M. Y. (1999). The conditions for tool use in primates: implications for the evolution of material culture. *Journal of Human Evolution*, *36*(6), 719-741.

Visalberghi, E. & Fragaszy, D.M. (1990). Do monkeys ape? In: Parker ST, Gibson KR (Eds.) *"Language" and intelligence in monkeys and apes: comparative and developmental perspectives.* (pp. 247 – 273) Cambridge, UK: Cambridge University Press

Westergaard, G. C., & Fragaszy, D. M. (1987). The manufacture and use of tools by capuchin monkeys (Cebus apella). *Journal of Comparative Psychology*, *101*(2), 159.

Whiten, A., Horner, V., & De Waal, F. B. (2005). Conformity to cultural norms of tool use in chimpanzees. *Nature*, *437*(7059), 737-740.

Wickham, H. (2016). *ggplot2: Elegant Graphics for Data Analysis*. Springer-Verlag New York. ISBN 978-3-319-24277-4, https://ggplot2.tidyverse.org.

Wilke, C. O. (2019). cowplot: Streamlined Plot Theme and Plot Annotations for 'ggplot2'. R package version 1.0.0. https://CRAN.R-project.org/package=cowplot

Wittig, R. M., & Boesch, C. (2003). Food competition and linear dominance hierarchy among female chimpanzees of the Tai National Park. *International Journal of Primatology*, *24*(4), 847-867.

Yamanashi, Y., Matsunaga, M., Shimada, K., Kado, R., & Tanaka, M. (2016). Introducing tool-based feeders to zoo-housed chimpanzees as a cognitive challenge: spontaneous acquisition of new types of tool use and effects on behaviours and use of space. *Journal of Zoo and Aquarium Research*, *4*(3), 147-155.
